# Truly pattern: Nonlinear integration of motion signals is required to account for the responses of pattern cells in rat visual cortex

**DOI:** 10.1126/sciadv.adh4690

**Published:** 2023-11-08

**Authors:** Giulio Matteucci, Rosilari Bellacosa Marotti, Benedetta Zattera, Davide Zoccolan

**Affiliations:** Visual Neuroscience Lab, International School for Advanced Studies (SISSA), Trieste 34136, Italy.

## Abstract

A key feature of advanced motion processing in the primate dorsal stream is the existence of pattern cells—specialized cortical neurons that integrate local motion signals into pattern-invariant representations of global direction. Pattern cells have also been reported in rodent visual cortex, but it is unknown whether the tuning of these neurons results from truly integrative, nonlinear mechanisms or trivially arises from linear receptive fields (RFs) with a peculiar geometry. Here, we show that pattern cells in rat primary (V1) and lateromedial (LM) visual cortex process motion direction in a way that cannot be explained by the linear spatiotemporal structure of their RFs. Instead, their tuning properties are consistent with and well explained by those of units in a state-of-the-art neural network model of the dorsal stream. This suggests that similar cortical processes underlay motion representation in primates and rodents. The latter could thus serve as powerful model systems to unravel the underlying circuit-level mechanisms.

## Teaser

Rat visual neurons encode global motion direction of composite visual patterns via nonlinear integration mechanisms.

## INTRODUCTION

Perceiving the velocity (i.e., motion direction and speed) of visual objects is critical to interact effectively with the environment. From a computational point of view, primary visual cortex can be thought as a bank of local moving-edge detectors. Upstream sensory areas face the nontrivial challenge of extracting object motion direction from the V1 representation. The output of a single localized edge detector is intrinsically ambiguous, since it reflects the projection of the global velocity vector onto the direction that is orthogonal to the orientation detected by the unit. Any information regarding the component of object motion that is parallel to such orientation is lost. Thus, when considered in isolation, the output of each of these edge detectors is compatible with infinite combinations of global directions and speeds and is therefore insufficient to fully specify the velocity of the underlying object. Only by combining (i.e., integrating) multiple local direction signals of this kind, global object direction and speed can be fully determined. This ambiguity is known in the neuroscientific literature as the “aperture problem” (*1*–*3*). Psychophysically, it can be appreciated by the fact that observers looking at a drifting object through a small aperture will perceive the edge seen through the aperture as always drifting in the perpendicular direction to the edge itself, irrespectively of the global direction of the object behind the aperture (*3*). If not handled properly by the visual system, the aperture problem would lead to illusory and inaccurate motion measurements (see movie S1).

In the brain of primates, motion integration is known to be achieved by pattern cells, which are abundant in monkey dorsal stream areas such as middle temporal (MT) (*4*–*11*) and medial superior temporal (MST) (*9*, *10*). The complementary class of cells is known as component cells—neurons that, behaving more like the local moving-edge detectors described above, are sensitive to the aperture problem. This class of neurons has been reported to be predominant in V1 (*4*, *6*, *9*, *10*) and widespread across multiple areas of the monkey visual cortex.

In rodents, only a handful of studies have investigated the distribution of pattern and component cells in mouse V1 (*12*, *13*) and bordering high-order visual areas (*14*). Such studies yielded contrasting results about the presence of pattern cells in V1: Two of them reported a small but consistent fraction of pattern units in this area (*12*, *13*), while another did not find any, reporting instead their presence in lateromedial (LM) and rostrolateral (RL) visual cortex (*14*).

Despite these contrasting findings on the neurophysiological front, recent work has provided compelling evidence about the causal involvement of V1 in mediating discrimination of motion direction of random dot fields in mice (*15*). Moreover, in a previous study, we have shown that rats can spontaneously perceive global motion direction of drifting plaids—i.e., composite patterns, made of two superimposed gratings drifting along different directions, which are typically used to distinguish pattern from component cells (*16*). In that study, rats trained to discriminate plaids drifting along opposite directions successfully generalized their discrimination to drifting gratings, thus displaying an ability consistent with the existence of pattern cells. However, rats trained with drifting gratings did not generalize to drifting plaids. With the help of computational modeling, we have hypothesized two alternate scenarios that could explain this finding [see (*16*) for details]. Which of these scenarios account for the behavioral findings critically depends on the relative impact of cross-orientation suppression on rat component and pattern cells, which is currently unknown.

**Fig. 1.**
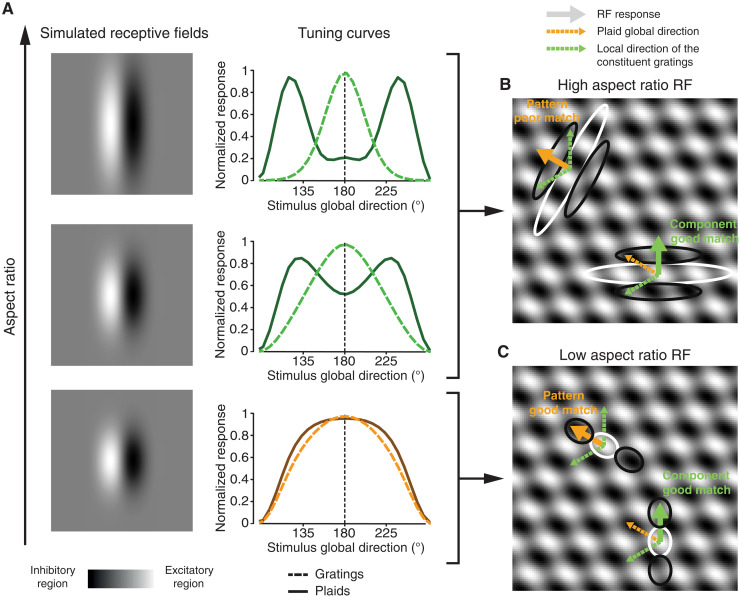
Illustration of how blobby, linear receptive fields can produce tuning consistent with the behavior of pattern cells. (**A**) Left: Spatial structure of the linear filters used to simulate receptive field (RFs) with progressively lower aspect ratio (from top to bottom) using Gabor functions. Right: Tuning curves showing the selectivity for grating and plaid direction (dashed and solid lines, respectively) of the linear filters shown on the left. (**B**) Cartoon of an elongated RF with high aspect ratio that poorly matches the local contrast features of a plaid drifting at 135° (orange arrow), made of two superimposed gratings drifting at 90° and 210° (green arrows). When the orientation of the RF is orthogonal to the local direction of one of the constituent gratings (bottom/right), there will be times at which the average luminance falling within the subunits of the RF matches their polarity, producing a strong response. If instead the RF is orthogonal to the global direction of the plaid (top/left), the average luminance falling within each RF subunit is approximately mid-gray, producing no response. The unit can thus signal the local direction of the grating (thick green arrow) but not the global direction of the plaid (thick orange arrow). (**C**) Cartoon of a blobby RF with low aspect ratio that matches well the local contrast features of the plaid [same stimulus as in (B)]. Regardless whether the RF is orthogonal to the local direction of the constituent gratings (bottom/right) or to the global direction of the plaid (top/left), there will be times at which the average luminance falling within the RF subunits matches their polarity, producing strong responses. The unit will thus respond similarly to gratings and plaids drifting along the same direction, yielding the pattern-cell tuning shown in (A) (bottom plot). Cartoons in (A) and (B) are inspired from (*17*).

More in general, the limited evidence about a hierarchical growth of pattern cells along the mouse putative dorsal stream, their overall paucity, and the fact that they have been found as early as in V1 raise the question of whether these neurons truly perform those nonlinear, integrative computations that are typical of primate pattern cells. As originally proposed by (*17*), a unit with a Gabor-like, linear RF can be tuned to the global motion direction of a plaid, thus displaying a pattern-like behavior, if the patches of local luminance in the plaid tightly overlap with the excitatory/inhibitory subfields of the cell’s RF (see cartoon in Fig. 1C). The shorter and wider (i.e., the “blobbier”) the RF subfields are (compare cartoons in Fig. 1, B and C), the more pattern-like the direction tuning of the cell will look like. Following (*17*), we quantitatively probed this scenario by simulating edge-detector units using Gabor filters with different aspect ratios and by measuring their responses to gratings and plaids (with a 120° cross-angle) drifting along 12 equi-spaced drift directions (Fig. 1A). Filters with high aspect ratio (top row) produced sharp tuning curves for both the grating (dotted line) and plaid (solid line) stimuli. Moreover, the tuning curve for the plaids peaked at ±60° (i.e., half plaid cross-angle) from the preferred direction of the gratings—the signature property of component cells. For filters with an intermediate aspect ratio (middle row), the tuning curves became broader and the two peaks of the curve obtained for the plaids partially overlapped, displaying a tendency to merge. Finally, for very low aspect ratios (bottom row), the further broadening of the tuning curves led to a complete merge of the two peaks of the curve obtained for the plaids into a single peak—thus yielding curves centered on the same (global) direction for both plaids and gratings. This shows how the defining property of pattern cells (i.e., similar tuning curves for gratings and plaids) could arise from purely linear spatial filters by virtue of their geometry (Fig. 1, B and C).

As already pointed out by (*13*), the mechanism illustrated above implies the possibility that pattern selectivity observed in rodent visual cortex could simply be the result of linear RFs with low aspect ratio and broad direction tuning. This scenario is consistent with the fact that, compared to mammals with higher visual acuity (*18*–*21*), rodent visual neurons have indeed broader tuning curves, preferences for lower spatial frequencies (SFs) (*22*–*24*), and RFs with a lower aspect ratio (*23*, *25*). In addition, although Palagina *et al*. (*13*) reported no substantial difference of average tuning broadness between pattern and component cells in mouse V1, they found that pattern responses are not cross-angle invariant (i.e., they change their pattern/component behavior depending on the angle between the two component gratings forming the plaid)—a signature of a possible dependence from RF geometry.

In light of these considerations, when investigating rodent visual cortex, the risk of misclassifying linear, non-integrative units (that should be properly considered as broadly tuned component cells) as pattern cells cannot be overlooked. The most direct way to test for this possibility is to reconstruct the linear receptive fields (RFs) of putative pattern cells and try to predict their plaid and grating responses on that basis. If the geometry of their RFs is the cause of their pattern-like tuning, the responses predicted by linear RFs should still be pattern-like. Conversely, if nonlinear, truly integrative mechanisms are at work, linear RFs should fail to produce pattern-like responses.

Our study was designed to (i) address this question about the nature of rodent pattern and component cells, (ii) assess their spatial tuning properties, and (iii) measure their abundance across rat V1 and extrastriate visual areas LM and RL. Finally, the entire data processing pipeline applied to the neuronal data was used to characterize the RF structure and tuning properties of the units of a state-of-the-art neural network model of the monkey dorsal stream (*26*). The network was also used to build predictive models of the tuning of rat pattern and component cells. This allowed a quantitative assessment of the level of sophistication of motion processing by rat visual cortical neurons and an indirect comparison with primate visual cortex.

## RESULTS

We performed extracellular recordings from visual areas V1, LM, and RL of anesthetized rats. The animals were presented with a battery of stimuli including (i) gratings and plaids (with a 120° cross-angle), drifting along 12 equi-spaced directions (from 0° to 330°) and presented at two different spatial and temporal frequencies (SFs = 0.02 and 0.04 cycles per degree (cpd); TFs = 2 and 6 Hz), and (ii) spatiotemporally correlated noise movies (see Materials and Methods). Grating and plaid responses were used to compute direction tuning curves and classify the recorded single units as pattern or component cells, based on the standard approach developed in cat and monkey studies (*4*, *5*, *11*, *21*, *27*), and also used in previous rodent studies (*12*–*14*). Noise movies were used to obtain a linear estimate of the spatiotemporal RF of each neuron (i.e., to find the best linear filter that approximated the stimulus-response function) by using the spike-triggered average (STA) technique (*28*–*30*).

We recorded a total of 447, 367, and 412 well-isolated single units from V1, LM, and RL, respectively. Among these neurons, 258 units in V1, 187 units in LM, and 184 units in RL were significantly responsive to gratings or plaids (see Materials and Methods) and were therefore included in the analyses described below. Following previous studies (*4*, *5*, *11*, *21*, *27*), “patternness” and “componentness” were quantified by computing the Fisher-transformed partial correlation between the observed responses to the plaids and the predicted responses to the same stimuli, as inferred from the observed responses to the gratings, assuming either an ideal pattern or component selectivity (these correlations are referred to as *Z*_p_ and *Z*_c_, respectively; see Supplementary Text for a definition). Direction-selective neurons with *Z*_p_ significantly higher than 0 and larger than *Z*_c_ were classified as pattern cells; vice versa, direction-selective units with *Z*_c_ significantly higher than 0 and larger than *Z*_p_ were classified as component cells (see Supplementary Text). Neurons that did not meet one of these requirements were labeled as unclassified.

**Fig. 2.**
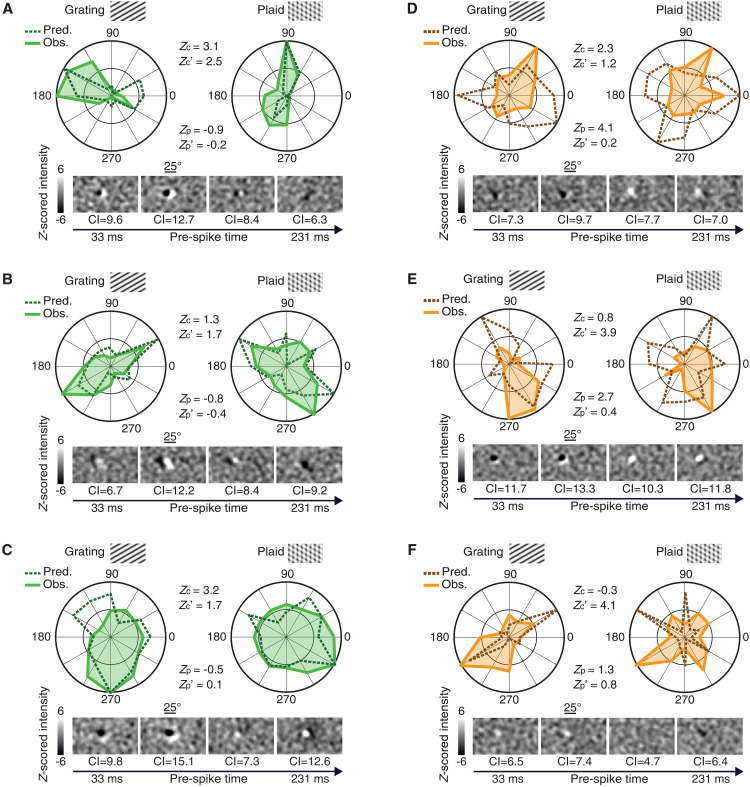
Examples of pattern and component cells recorded in areas V1, LM, and RL. Each panel depicts the observed tuning curves (i.e., normalized average response for each stimulus direction) for gratings (left; solid line) and plaids (right; solid line), as well as the tuning curves predicted by an LN model based on the spatiotemporal filter estimated via STA (dashed lines). The pairs of values *Z*_c_ and *Z*_p_ and *Z*_c_′ and *Z*_p_′ are the component and pattern indexes computed for the observed and predicted tuning curves, respectively. The temporal sequence of STA images at different time lags preceding spike generation is also shown (bottom), along with the CI values that quantify the sharpness of each STA filter. In every STA image, each pixel intensity value was independently *z*-scored, based on the null distribution of STA values obtained through a permutation test (see Supplementary Text). A gray-scale map was used to visualize the resulting *z*-scored values within the [−6, +6] range (see scale bars). (**A**) Sharply tuned component cell from V1, correctly predicted by the LN model as component. (**B**) Sharply tuned component cell from LM, correctly predicted by the LN model as component. (**C**) Broadly tuned component cell from V1, correctly predicted by the LN model as component. (**D**) Sharply tuned pattern cell from LM, incorrectly predicted by the LN model as unclassified. (**E** and **F**) Sharply tuned pattern cells from LM, incorrectly predicted by the LN model as component.

Figure 2 shows the tuning of a few example neurons recorded from the three targeted areas and classified as either component or pattern cells. For each unit, the figure reports (i) the normalized tuning curve as a function of the direction of the stimulus (either a grating, left, or a plaid, right), when presented at the most effective SF and TF (solid lines); (ii) a sequence of STA images, computed at progressively larger time lags from the firing of an action potential, showing the spatiotemporal evolution of the RF structure; and (iii) the tuning curves (dashed lines) predicted by a linear-nonlinear (LN) model of stimulus-response mapping using the spatiotemporal filter estimated via the STAs (*28*–*30*). The figure also reports the values of the metrics used to quantify the response properties of the neurons, i.e., (i) the patternness and componentness indexes for both the observed (*Z*_p_ and *Z*_c_) and predicted (*Z*_p_′ and *Z*_c_′) tuning curves and (ii) the contrast index (CI) used to quantify the sharpness of the STA images (*25*, *31*).

Figure 2A shows a typical V1 component cell displaying clear direction selectivity. When probed with the gratings, the unit’s direction tuning curve featured a well-defined peak in the 150° to 180° range. By contrast, when measured with the plaids, the curve displayed two peaks at about ±60° with respect to the direction of the preferred grating. This inconsistency between the peak responses obtained for gratings and plaids is what made the unit a component cell, i.e., a poor global motion detector. The neuron was not sensitive to the actual direction of the plaid, but to the direction of its constituent gratings—only when the latter aligned with the preferred grating direction, the unit fired vigorously. The figure also shows how STA returned images with large CI values containing crisp, Gabor-like RFs made of two flanking lobes (one excitatory and one inhibitory), aligned along an axis that was orthogonal to the unit’s preferred orientation, and with a phase that gradually shifted over time, consistently with the strong direction selectivity of the neuron. The STA images yielded a good approximation of the unit’s spatiotemporal RF, as demonstrated by the close match between the measured tuning curves and those predicted by the LN model for both the grating and plaid responses (compare solid and dashed lines). As a result, *Z*_c_ was considerably larger than *Z*_p_ for both the observed and predicted tuning curves, yielding a classification of the cell as component in both cases. A similar behavior can be observed for two other example cells—one recorded in LM, having a sharp orientation tuning curve when tested with gratings (Fig. 2B), and another one, recorded in V1, with broader tuning (Fig. 2C). In both cases, the tuning curve obtained with the plaids featured two peaks, roughly at ±60° with respect to the direction of the preferred grating, and the spatiotemporal RF was well captured by STA, yielding, again, multi-lobed, Gabor-like filters (with large CI values) that accurately predicted grating and plaid responses via the LN model. As a result, *Z*_c_ was larger than *Z*_p_ for both the observed and predicted curves, indicating that both units were component cells and were correctly predicted as such by the LN model.

A different behavior can be observed for the three example LM neurons shown in Fig. 2, D to F (orange curves). In all cases, the units were narrowly tuned for a specific grating direction, and such preference was maintained when tested with the plaids, yielding, for both stimulus classes, highly consistent, sharply tuned curves. This is the typical tuning expected for pattern cells, as confirmed by the larger magnitude of *Z*_p_, as compared to *Z*_c_. This behavior was not captured by the LN model. In the case of the first cell (Fig. 2D), STA returned a “blobby” RF with a main, dominant lobe and lower CI (on average, across frames), as compared to the component cells shown in Fig. 2 (A to C). As a result, the STA-based LN model only partially accounted for the tuning for grating direction and fully failed to predict the tuning for plaid direction (compare solid and dashed lines). In the case of the second cell (Fig. 2E), STA images with high contrast (CI) were obtained across almost the entire spatiotemporal evolution of the RF. The resulting LN model successfully predicted the tuning of the unit for grating orientation, although not for direction. Critically, however, the LN model failed to account for the tuning for plaid direction, yielding a predicted tuning curve, with peaks at about ±60° with respect to the direction of the preferred grating, consistent with the behavior of a component rather than a pattern cell. A similar behavior was observed for the third cell (Fig. 2F), whose RF, as recovered by STA, despite being poorly structured and with rather low CI, succeeded at roughly capturing the tuning of the unit for grating orientation (but, again, not for direction). However, as for the previous neuron, the LN model failed to predict the tuning for plaid direction, returning a curve that was inconsistent with the behavior of a pattern cell, having peaks at about ±60° with respect to the preferred grating direction. As a result, although the three neurons in Fig. 2 (D to F) were classified as pattern cells, based on the observed responses to gratings and plaids, none of them retained such classification when their predicted responses via the LN model were considered—based on such predictions, the cell shown in Fig. 2D fell in the unclassified category, while the cells shown in Fig. 2 (E and F) were classified as component.

### A small fraction of pattern cells coexists with component cells in cortical areas V1 and LM

**Fig. 3.**
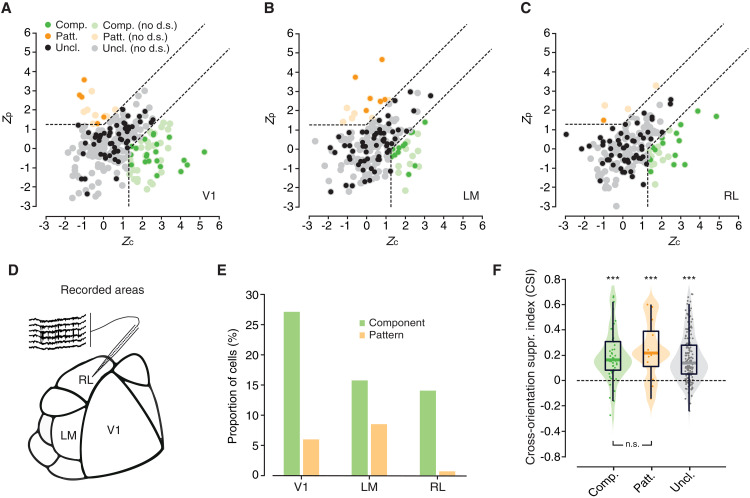
Distribution of pattern and component cells in areas V1, LM, and RL. (**A** to **C**) Scatter plots showing the distributions of the pairs of observed *Z*_p_ and *Z*_c_ indexes for the cells recorded in the three targeted areas. Light-colored dots represent units not meeting the direction selectivity criterion (i.e., DSI > 0.33); dark-colored dots represent units meeting such criterion. Dashed lines are decision boundaries in the *Z*_p_/*Z*_c_ plane that distinguish regions where cells are labeled as component (in green; bottom-right corner), pattern (in orange; top-left corner), or unclassified (in gray; central area). (**D**) Schematic map of rat visual cortex showing the anatomical locations of V1, LM, and RL. (**E**) Percentage of component cells (green bars) and pattern cells (orange bars) recorded in V1 (left), LM (center), and RL (right). (**F**) Distributions of cross-orientation suppression index (CSI) values across area-pooled populations of component (left; green), pattern (center; orange), and unclassified (right; gray) units. All medians were significantly larger than zero (***P < 0.001, Wilcoxon test). The medians of the component and pattern cells’ pools were not statistically different from each other (*P* > 0.05, Wilcoxon test). n.s., nonsignificant.

The example neurons shown in Fig. 2 demonstrate that rat visual cortical areas do contain neurons that can robustly be classified as either pattern or component cells. Figure 3 (A to C) reports the incidence of these cell types in the investigated areas, by plotting, for each unit, the pair of *Z*_p_ and *Z*_c_ indexes (the anatomical location of the areas over the cortical surface is indicated in Fig. 3D). The boundaries in the figures indicate regions of the *Z*_p_/*Z*_c_ plane where one of the indexes is sufficiently larger than zero, as well as sufficiently larger than the other one, for a cell to be classified as either component (in green; bottom-right corner) or pattern (in orange; top-left corner). All other neurons were considered unclassified (in gray; central region). In addition, neurons were labeled according to whether they were sufficiently direction tuned (darker shades), i.e., with a direction selectivity index (DSI) > 0.33—an additional requirement that allows classifying a unit as a component (or pattern) direction-selective cell (see Materials and Methods).

As shown in Fig. 3E, our recordings yielded a small amount of pattern cells in V1 (6% of 78 units) and a slightly larger fraction in LM (9% of 76 units). By contrast, we found in RL only a single pattern cell. As for component cells, their incidence was larger in V1 (27% of 78 units) than in RL (21% of 73 units) and LM (16% of 76 units). Finally, consistently with previous rodent studies (*12*–*14*), the largest fraction of single units included in the analysis fell into the unclassified category (67% in V1, 76% in LM, and 77% in RL). All these percentages refer to the pools of responsive units meeting the direction selectivity criterion in each area.

Overall, these results confirm that, also in rats, as previously observed in mice, motion-sensitive neurons exist, which can be classified as either component or pattern cells, according to the criteria commonly adopted in the monkey literature, although the incidence of pattern cells is way lower than in primate dorsal stream areas (*9*, *10*). In addition, we observed a small increase in the proportion of direction-selective pattern cells from V1 to LM, paralleled by a decrease in the proportion of component cells. Although such a trade-off in the relative abundance of the two cell types did not reach statistical significance (*P* = 0.1870, χ^2^ test; a tendency toward significance was observed when proportions were computed over the whole pool of responsive units; *P* = 0.1), we nevertheless found a significant increase of the average value of the pattern index (PI = *Z*_p_ − *Z*_c_, a commonly used scalar metric of patternness) from V1 (PI = −1.64 ± 0.31) to LM (PI = −0.57 ± 0.40; *P* < 0.05, unpaired *t* test). Together, these observations are suggestive of a shallow, hierarchical buildup of global motion detectors from V1 to LM.

Next, we checked the extent to which these cell types are affected by cross-orientation suppression, given the relevance of this property to probe alternative perceptual models of motion integration in rats (*16*). To measure this phenomenon, we computed a cross-orientation suppression index (CSI), which quantifies the relative amount of suppression (or facilitation) of neuronal firing when a unit is tested with its preferred plaid stimulus, as compared to its preferred grating stimulus (see Materials and Methods): A positive value indicates suppression (i.e., larger peak response for gratings than for plaids), while a negative value indicates facilitation (i.e., larger peak response for plaids than for gratings). As shown in Fig. 3F, the CSI values for both component and pattern cells were shifted toward positive values (green and orange distributions), with the medians of the two populations being significantly higher than zero (*P* < 0.001, Wilcoxon test) but not statistically different from each other (*P* > 0.05, Wilcoxon test). The same trend was observed for the pool of unclassified units (gray distribution). Thus, for both component and pattern cells, plaids tended to elicit lower responses than gratings. The implication of this result for understanding the perceptual mechanisms underlying motion integration in rats is examined in Discussion.

### Linear spatiotemporal RFs are sharper and more structured for component than for pattern cells

The main goal of our study, beside assessing whether pattern cells are present in rat visual cortex, was to understand whether the tuning of these units can properly be ascribed to nonlinear integrative mechanisms or, instead, is mainly the result of linear filtering via blobby RFs (see Introduction and Fig. 1). To address this question, we measured the time evolution of spatial RFs at 10 progressively longer time lags from spike generation using STA analysis (see examples in Fig. 2). We then tested the hypothesis that STA better captures the RFs of component than pattern cells, as expected if the stimulus-response relationship was more nonlinear for the latter.

**Fig. 4.**
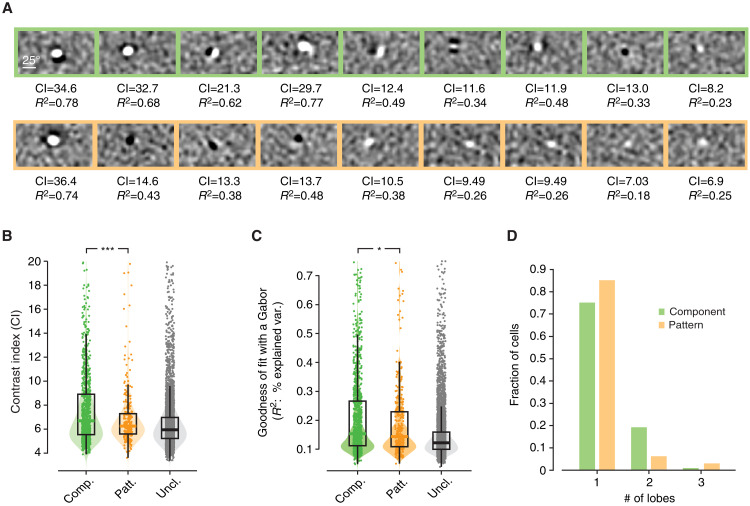
Component cells have sharper and more Gabor-like, linear RFs than pattern cells. (**A**) Representative examples of STA images obtained for nine component cells (top, green frames) and nine pattern cells (bottom, orange frames). Each image is shown along with the corresponding CI and a metric (*R*^2^) that quantifies the GOF with a Gabor function. For each neuron, the RF shown here is taken at the time before spike generation when the number of distinct subfields (lobes) was the largest. (**B**) Distributions of CI values of the STA images obtained for the area-pooled populations of component (left, green), pattern (center, orange), and unclassified (right, gray) units at all tested (10) time lags from spike generation. All units in the proper quadrants of the *Z*_c_/*Z*_p_ plane, with no constraint on direction selectivity, were included in this analysis (i.e., all green, orange, and gray dots of Fig. 3, A to C, were used, no matter whether light or dark). The median CIs of component and pattern cells were significantly different according to a Wilcoxon test (***P = 0.003). (**C**) Same as in (B), but for the distributions of *R*^2^ values (assessing the GOF with a Gabor function), whose medians were significantly different, for the component and pattern cells, according to a Wilcoxon test (*P = 0.03). (**D**) Distribution of the number of distinct lobes in the STA image (binarized at 3.5σ) with the largest CI obtained for the component (green bars) and pattern (orange bars) cells shown in Fig. 3, A to C, [as in (B) and (C), no constraint was imposed on the level of direction selectivity]. The distributions were statistically different according to a χ^2^ test (*P* = 0.047).

As illustrated by the example units of Fig. 2 and by the additional examples of Fig. 4A, component cells do appear to have RFs that are somewhat sharper (i.e., with larger contrast, as compared to the background noise) than those of pattern cells, and closer to Gabor functions containing at least two lobes. In Fig. 4, following an approach we already adopted in previous studies (*25*, *31*), we statistically quantified this comparison, by plotting the distributions of (i) the CI values obtained for the STA images in the two populations of component and pattern cells at all tested (10) time lags from spike generation (Fig. 4B), (ii) the goodness of fit (GOF) of all these STA images with Gabor functions (i.e., fraction of explained variance; Fig. 4C), and (iii) the number of distinct, prominent lobes in the sharpest STA image (i.e., image with largest CI) obtained for each cell (Fig. 4D).

Note that, to increase statistical power, in this analysis, we counted as component and pattern cells all units in the proper quadrant of the *Z*_c_/*Z*_p_ plane, with no constraint on direction selectivity (this corresponds to all orange and green dots in Fig. 3, A to C, no matter whether light or dark). This yielded a total of 98 component and 30 pattern cells, each contributing 10 STA images. As illustrated in Fig. 4, B and C,, both the median CI and GOF were slightly but significantly larger for component than for pattern cells (*P* = 0.003 and *P* = 0.03, Wilcoxon test). The lobe count distributions were also statistically different (*P* = 0.047, χ^2^ test), with a larger proportion of pattern than component cells having RFs with just a single prominent lobe (Fig. 4D).

Overall, this indicates that STA was relatively less successful at capturing the spatial structure of RFs in the case of pattern cells. This does not mean that it completely failed at doing so. As shown by the example neurons in Figs. 2, D to F, and 4A, STA did often return, even in the case of pattern cells, some well-structured RFs. However, the overall lower contrast, “Gaborness,” and complexity of the RFs of pattern cells suggest a more prominent contribution of nonlinear terms (not captured by STA) in establishing their stimulus-response mapping, as compared to component cells. The crucial question is the extent to which the linear RFs inferred via STA are able to account for the pattern and component nature of the two populations.

### Response predictions based on linear RFs yield an incorrect classification of pattern cells

For the example component cells of Fig. 2, A to C,, the observed tuning curves for both grating and plaid direction (solid green lines) were generally well matched by the curves predicted on the base of the linear RFs inferred via STA (dashed green lines). As a result, the units retained their classification as component cells when the *Z*_c_ and *Z*_p_ indexes were computed on the predicted curves. By contrast, for the example pattern cells of Fig. 2, D to F, (solid orange lines), the predictions based on linear RFs (dashed orange lines) accounted at most for the tuning for grating orientation but failed to capture the tuning for plaid direction. This led to a misclassification of two of the units as component when the *Z*_c_ and *Z*_p_ scores were computed on the predicted curves. We checked whether this phenomenon was statistically consistent at the population level.

Predictions obtained using STAs as linear filters in an LN model work at best when the SF of the stimulus fed to the model matches the dominant SF of the STA itself (i.e., the spatial scale of the excitatory and inhibitory lobes in the STA). In our case, we empirically observed that the spatial scale of the RFs estimated via STA better matched the gratings with SF = 0.02 cpd (see fig. S1A). We therefore computed predictions for responses to gratings and plaids with such SF. To guarantee the consistency with the analysis shown in Fig. 3 (where the *Z*_p_ and *Z*_c_ indexes were computed for stimuli at the most effective SF), we restricted the pool of neurons included in this analysis to units that were consistently classified as either component or pattern at both SF = 0.04 and SF = 0.02. This reduced the overall pools of component and pattern cells (merged across the three areas) from, respectively, 98 and 30 (i.e., all green and orange dots across Fig. 3, A to C) to 58 and 17.

**Fig. 5.**
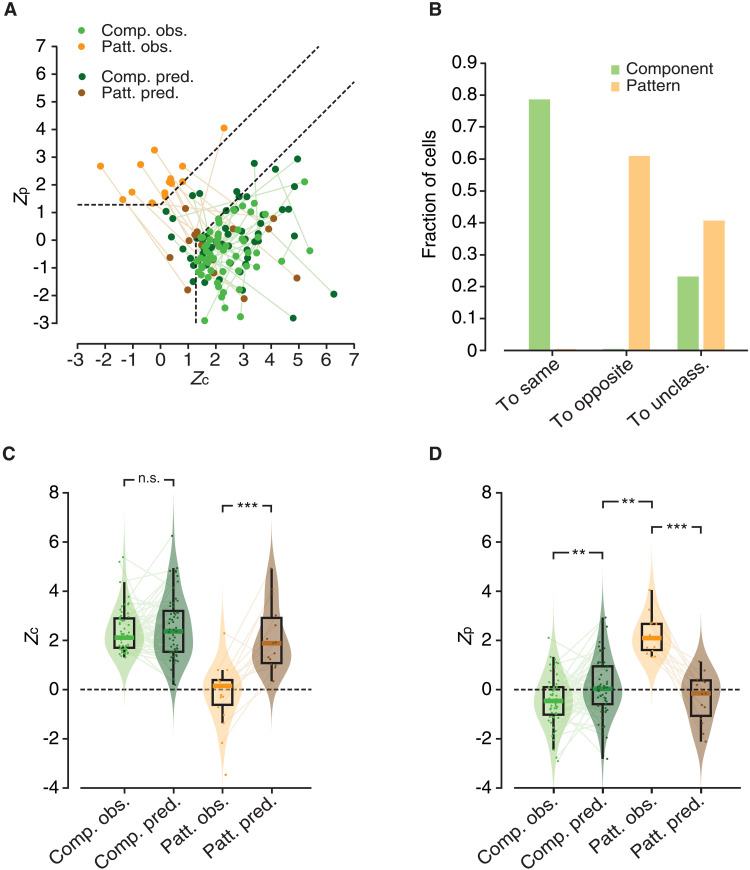
Linear RFs do not account for global motion selectivity of pattern cells. (**A**) Scatter plot showing the distributions of the pairs of observed *Z*_p_ and *Z*_c_ indexes for pattern (light orange) and component cells (light green) recorded across V1, LM, and RL together with the values of the same indexes (dark orange and green dots), when computed on the tuning curves predicted using the spatiotemporal RFs that were estimated via STA. The lines connect the observed and predicted pairs of index values to highlight the displacement in the *Z*_p_/*Z*_c_ plane for each unit. Same layout and color code as in Fig. 3, A to C. (**B**) Bar plot reporting the fraction of units (pattern cells in orange and component cells in green) that preserved their original classification (left; “To same” label), switched to the opposite class (center; “To opposite” label), or landed in the unclassified region (right: “To unclass.” label), when the *Z*_p_ and *Z*_c_ indexes were computed on the predicted direction tuning curves. (**C**) Distributions of observed (light colors) and predicted (dark colors) *Z*_c_ values for the two populations of component (green) and pattern (orange) cells. (**D**) Distributions of observed (light colors) and predicted (dark colors) *Z*_p_ values for the two populations of component (green) and pattern (orange) cells. In (C) and (D), the statistical comparison between the medians of the observed versus predicted distributions for the same neuronal populations was performed using a paired Wilcoxon test (**P < 0.01; ***P < 0.001). In (D), the statistical comparison between the median of the predicted *Z*_p_ indexes for the component cells versus the median of the observed *Z*_p_ indexes for the pattern cells was carried out using an unpaired Wilcoxon test (**P < 0.01).

Figure 5A illustrates the distribution of these two populations in the *Z*_c_/*Z*_p_ plane (light green and light orange dots) and shows how the pairs of *Z*_c_ and *Z*_p_ values obtained for each unit changed when it was computed on the direction tuning curves predicted by the LN model (dark green and dark orange dots). The difference between the two populations was striking. While most component cells remained in the “component” region of the plane and those that changed category became at most unclassified, none of the pattern cells retained its classification and many of them ended up being classified as component. This is better quantified by the bar plot in Fig. 5B, comparing which fraction of units in the two populations retained its original classification (e.g., component remaining component), which fraction switched to the opposite class (e.g., component becoming pattern), and which fraction became unclassified, based on their responses to gratings and plaids as predicted by the LN model. The two distributions were radically (and significantly; *P* = 5.9 × 10^−11^, χ^2^ test) different, thus testifying the inability of the linear RFs estimated via STA to capture the patternness of pattern cells.

To better understand the cause of this phenomenon, we separately plotted for the two cell populations the distributions of the *Z*_c_ and *Z*_p_ indexes, as computed based on the observed responses (light shading) and the predicted ones (dark shading). In the case of component cells (Fig. 5C, green shadings), *Z*_c_ remained very stable, with no detectable difference between the medians of the observed and predicted values (*P* = 0.84, paired Wilcoxon test). By contrast, for pattern cells (Fig. 5C, orange shadings), the median *Z*_c_ increased substantially (and significantly; *P* < 0.001, paired Wilcoxon test): from close to zero (as it had to be, given the classification of these units as pattern cells based on their observed responses) to close to 2 (i.e., close to the value observed for component cells). This, together with the trend observed for *Z*_p_ (see below), explains why many pattern cells switched their status from pattern to component when the responses of the LN model were considered (see Fig. 5, A and B).

A similar result was found for the *Z*_p_ index (Fig. 5D). In the case of component cells (green shadings), its median remained quite stable, although it was significantly closer to zero for the predicted than for the measured responses (*P* = 0.005, paired Wilcoxon test). However, this increase was marginal and the index remained substantially and significantly lower than the large positive values (close to 2) observed for pattern cells (compare the dark green to light orange distributions; *P* = 0.006, unpaired Wilcoxon test). This explains why most component cells retained their classification when *Z*_c_ and *Z*_p_ were computed based on their predicted responses (Fig. 5, A and B). Pattern cells displayed instead a very different behavior (orange shadings). The median *Z*_p_ dropped to zero when computed using the predicted responses of the LN model, being substantially lower than the value (close to 2) obtained for the observed responses (*P* < 0.001, paired Wilcoxon test). This explains why none of the pattern cells retained its pattern status when the predicted responses were considered (see Fig. 5, A and B).

Overall, this analysis shows that linear RFs inferred via STA are not good predictors for the global motion sensitivity of pattern cells, when used as linear filters in an LN encoding model. This strongly suggests that the tuning of pattern cells is the result of nonlinear integration of local direction signals (*8*, *32*).

### The incidence of complex cells is larger within the pattern than the component cells’ subpopulation

The nonlinear processes underlying the tuning of pattern cells are by no means the only ones that are thought to be at work in visual cortex. Another form of nonlinearity is the one by which complex cells integrate the inputs from position-sensitive simple cells to acquire their translation (or phase) invariance (*33*). It can be tempting to equate this nonlinear pooling to the one underlying the tuning of pattern cells—i.e., to equate simple to component cells and complex to pattern cells. However, the input signals integrated by the two classes of nonlinear units are very different. Complex cells should pool over presynaptic edge detectors (simple cells), all having the same orientation preference but slightly offset RF positions (*33*). Pattern cells should instead pool over edge detectors spanning a variety of direction preferences but having similar RF positions (*8*, *32*). To our knowledge, in the monkey literature, there is no systematic study of the co-occurrence of these two forms of nonlinearity. Here, we sought to quantify it for our recorded neuronal population.

To this end, we relied on the computation of a modulation index (MI; see Materials and Methods), which quantifies the extent to which the neuronal response of a unit is modulated at the TF of its preferred grating (*25*, *31*, *34*). This index measures the difference between the power of the response at the stimulus frequency and the average of the power spectrum in units of its standard deviation (SD). It is similar to the traditional F1/F0 ratio used in the monkey literature (*35*), but, being MI a standardized metric, it yields a statistically more robust classification of units as simple or complex [see (*31*) for a thorough discussion]—values larger than 3 can be interpreted as the signature of strong modulation (as typical of phase-sensitive simple cells), while values lower than 3 indicate poor modulation (as expected from phase-tolerant complex cells).

**Fig. 6.**
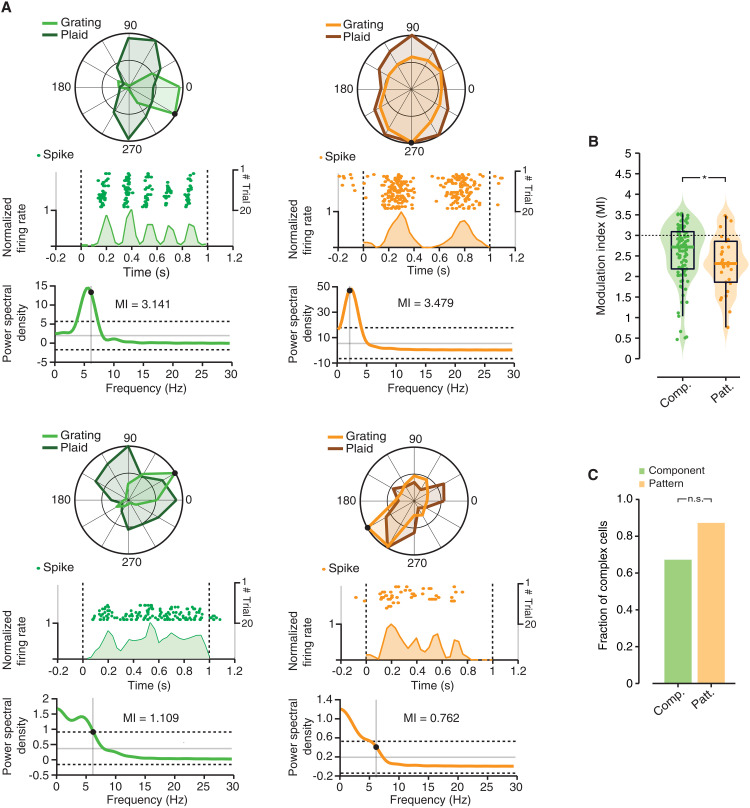
Phase invariance of component and pattern cells. (**A**) Response dynamics of two example component cells (left, green) and pattern cells (right, orange), following presentation of the most effective grating. Each panel depicts (i) the observed, normalized tuning curves for gratings (light color; the black dot indicates the response to the preferred grating) and plaids (dark color); (ii) the raster plot, with the times at which individual action potentials were fired across repeated presentations of the most effective grating, as well as the resulting peristimulus time histogram (PSTH), computed in 10-ms-wide time bins, which reports the normalized, trial-averaged firing rate of the neuron during stimulus presentation (stimulus onset and offset are marked by the vertical dashed lines); and (iii) the power spectrum of the PSTH, with its mean (horizontal gray line), its mean ± SD (dashed lines), and the TF of the grating (vertical gray line; the black dot marks the value of the power spectrum at the stimulus frequency). The value of the MI (defined in Materials and Methods) is also reported for each example cell. (**B**) Distributions of MI values for the populations of component (in green) and pattern (in orange) cells, as computed from the response to the most effective grating for each unit. The dashed line indicates the conventional threshold to distinguish simple (MI > 3) from complex cells (MI < 3). The median MI was slightly but significantly higher for component than for pattern cells (*P* < 0.05, Wilcoxon test). (**C**) Bar plot reporting the fraction of cells in each population being classified as complex cells (i.e., having MI < 3). Although this fraction was larger for pattern than for component cells, the difference did not reach statistical significance (*P* > 0.05, χ^2^ test).

Figure 6A shows the behavior of two example component (left) and two example pattern (right) cells. In addition to the tuning curves obtained for the grating and plaid stimuli, each panel reports the dynamic (raster plot and peristimulus time histogram; see figure legend for details) and the power spectrum of the response to the most effective grating. For the cells in the top row, the response was strongly modulated at the TF of the stimulus (6 and 2 Hz, respectively, for the component and pattern cell). As a result, the value of the power spectrum at the TF of the stimulus (black dot) was very close to the peak, and its magnitude was more than 3 SDs above the mean of the power spectral density. This yielded large MI values (3.14 and 3.48, respectively, for the component and pattern cell), which are consistent with the tuning of a simple cell. By contrast, the responses of the cells in the bottom row were way more stable over time. The value of the power spectrum at the TF of the stimulus was far from the peak (which was centered on the lowest measurable frequency) and had a magnitude that was close to 1 SD above the mean of the power spectral density. The resulting MI values were much smaller than 3 (1.10 and 0.76, respectively, for the component and pattern cell) and were consistent with the tuning of a complex cell.

These examples illustrate how, in our recorded populations, both component and pattern cells could be found that displayed spatial integration properties consistent with either a simple-like or a complex-like behavior. At the population level, pattern cells had a slightly but significantly lower median MI than component cells (2.31 versus 2.71; *P* = 0.04931, Wilcoxon test; Fig. 6B). Consistently, a larger fraction of pattern cells was classified as complex, as compared to component cells (~86% versus ~69%; Fig. 6C), although the difference did not reach significance (*P* = 0.1, Fisher exact test). This shows a general tendency of the two forms of nonlinearity (the one underlying tuning for global motion direction and the one at the base of translation invariance) to co-occur in rat visual cortical areas. At the same time, one kind of nonlinearity does not necessarily imply the other, as expected for two computations that are critical for two different processing streams (see the Discussion).

### The units of DorsalNet, a neural network model of the dorsal stream, display motion processing properties that are consistent with those of rat component and pattern cells

**Fig. 7.**
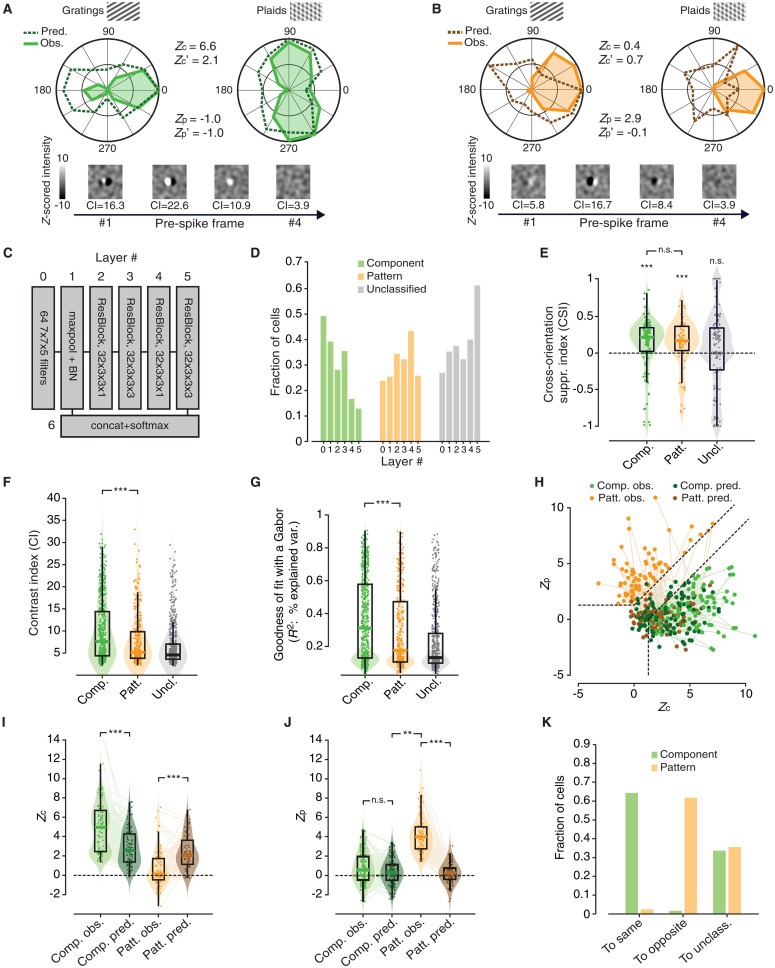
The units of DorsalNet display tuning properties that are highly consistent with those observed for rat visual cortical neurons. (**A** and **B**) Examples of DorsalNet units displaying the typical tuning of a component cell (A) and of a pattern cell (B). Same layout and color code used in Fig. 2. (**C**) Schematic of DorsalNet layer structure. (**D**) Fraction of component (green bars), pattern (orange bars), and unclassified (gray bars) units from layer #0 to layer#5 of DorsalNet (compare to Fig. 3E). (**E**) Distribution of CSI values for component (green), pattern (orange), and unclassified (gray) units, pooled across all DorsalNet layers (same layout, color code, and statistical analysis as in Fig. 3F). (**F** and **G**) Distribution of CI and *R*^2^ values for component (green), pattern (orange), and unclassified (gray) units (same layout, color code, and statistical analysis as in Fig. 4, B and C). (**H**) Scatter plot showing (i) the distribution of observed *Z*_p_ and *Z*_c_ indexes for pattern (light orange) and component (light green) units, pooled across all DorsalNet layers, and (ii) the values of the same indexes (dark orange and green), as computed for the tuning curves predicted using the spatiotemporal RFs that were estimated via STA (compare to Fig. 5A). (**I** and **J**) Distributions of observed (light colors) and predicted (dark colors) *Z*_c_ and *Z*_p_ values for the populations of DorsalNet units classified as component (green) and pattern (orange; same layout, color code, and statistical analysis as in Fig. 5, C and D). (**K**) Bar plot showing the fraction of DorsalNet units that preserved their original classification, switched to the opposite class, or became unclassified (same layout and color code as in Fig. 5B), when the *Z*_p_ and *Z*_c_ indexes were computed on the predicted direction tuning curves. The analyses in (E) to (K) refer to units of the same class, pooled across all DorsalNet layers, respectively.

The results shown in the previous sections suggest that a fraction of rat visual cortical neurons encode global motion direction of complex visual patterns via integrative, nonlinear processes that are consistent with those thought to be at work at the higher stages of the monkey dorsal stream (*8*, *9*). To further test the extent to which the properties of rat component and pattern cells are consistent with the existence of a functional motion processing hierarchy, we carried out a comparison with DorsalNet, a state-of-the-art model of the monkey dorsal stream (*26*). DorsalNet is a six-layer three-dimensional (3D) convolutional neural network (see Fig. 7C) that has been trained with the self-supervised learning objective of predicting the self-motion parameters of a virtual agent moving in a simulated environment from its own visual input. As a result of training, the units of the network developed a tuning for visual motion that can explain visual responses in a database of neural recordings from the primate dorsal stream better than many other computational models of motion processing (*26*). This makes DorsalNet the current best-in-class in silico model of the dorsal stream.

In our analysis, we used the pretrained DorsalNet model provided by (*26*), and we fed it with gratings and plaids drifting along the same 12 directions used in our recordings, as well as with spatiotemporally correlated noise movies derived from those used in our experiments. We then measured the responses (i.e., the activations) to these stimuli of all the units at the center of the convolutional map in the output layer of each of the six blocks of the network (Fig. 7C). Finally, we analyzed the tuning properties of the sampled units by using the same data processing pipeline applied to the neuronal data.

As already reported by (*26*), many units across the layers of the network displayed sharp direction selectivity, which, in some case, was consistent with the tuning of a component cell, while, in some other, with the tuning of a pattern cell. The example tuning curves reported in Fig. 7 (A and B) illustrate such cases, along with the sequences of STA images showing the inferred spatiotemporal structure of the units’ RFs (a striking similarity with the example component and pattern cells displayed in Fig. 2 can be noticed). As expected from a dorsal-like motion processing hierarchy, the proportions of component and pattern units traded off across the ResNet blocks of the network, with the former sharply decreasing from layer 0 to layer 5 and the latter smoothly increasing (compare the green to the orange bars in Fig. 7D), although both kinds of units coexisted in all layers. Both the component and pattern units responded less strongly to plaids than to gratings, showing, on average, CSI values significantly greater than zero (Fig. 7E; component: *P* = 0.0001, unpaired *t* test; pattern: *P* = 0.001, Wilcoxon test) but not statistically different from each other (*P* > 0.05, Wilcoxon test), as already observed for rat visual cortical neurons (Fig. 3F).

We found that, also in DorsalNet, the spatiotemporal RFs of component units (as inferred via STA) were sharper (i.e., with higher contrast; Fig. 7F; *P* = 4.2 × 10^−7^, Wilcoxon test), better approximated by Gabor filters (Fig. 7G; *P* = 4.1 × 10^−6^, Wilcoxon test), and better capable to predict the observed *Z*_p_ and *Z*_c_ values (Fig. 7, H to J; see legend for a statistical comparison of observed and predicted *Z* values), and, therefore, to preserve the units’ classification (Fig. 7K; *P* = 1.0 × 10^−300^, χ^2^ test), as compared to the RFs obtained for pattern units. All these trends matched strikingly well those observed for rat component and pattern cells (compare to Figs. 4 and 5). In particular, the consistency between the properties of pattern cells in rat visual cortex and those of pattern units in DorsalNet strongly supports the conclusion that the former are the result of nonlinear computations aimed at integrating local motion signals into pattern-invariant representations of global motion direction.

We also checked whether the spatial tuning of DorsalNet units displayed any tendency to become more phase-invariant across the layers and whether such tendency was stronger for the population of pattern than component units. As for the case of rat visual cortical neurons (Fig. 6A), we found examples of component and pattern units in DorsalNet that displayed either a simple-like or a complex-like tuning (fig. S2A). Also in the network, pattern units had a lower median MI than component units, but this difference was much smaller than for rat neurons (compare to Fig. 6B) and not significant (2.86 versus 2.99; *P* = 0.3565, Wilcoxon test; fig. S2B). The same applied to the fraction of complex units in the two populations (fig. S2C). The MI, however, changed considerably across the depth of the network (fig. S2D), dropping from being slightly above 3 in the first two layers (consistently with simple-like tuning) to values between 2 and 2.8 in the following layers (consistently with complex-like tuning). This trend was observed across all three populations of component, pattern, and unclassified units, with the fraction of simple cells dropping from 60 to 100% in the first two layers to 0 to 40% in the following ones (fig. S2E). Overall, this result reinforces the intuition that the nonlinear pooling mechanisms underlying the emergence of pattern cells and complex cells are independent processes that can coexist in hierarchical image-processing systems, but one does not necessarily imply the other (see Discussion).

### The tuning of rat pattern and component cells is well accounted for by the activations of DorsalNet pattern and component units

The comparison with DorsalNet carried out in the previous section was at level of emergent properties. That is, we asked whether prominent differences between the tuning properties of the populations of pattern and component DorsalNet units could be found that were consistent with those observed in rat visual cortex—discovering, in most cases, a very good match. We have successfully applied this approach in previous comparisons of processing along the rat ventral stream and deep neural networks for image classification (*25*, *36*). Its main advantage is that it allows assessing the extent to which two processing systems share fundamental principles underlying the reformatting of visual information, without the need of enforcing the fitting of one system to the other. An alternative and very popular approach consists in directly modeling the tuning of visual cortical neurons using, as regressors, the activations of the units of a deep neuronal network that has been fed with the same visual stimuli used to probe the neurons. This approach has been extremely successful in capturing the tuning of neurons at the highest stages of the monkey ventral stream, such as the inferotemporal cortex (*37*–*39*).

To complement the analyses reported in the previous section, we applied this second approach and we modeled the tuning of rat pattern and component cells using the activations of DorsalNet units (referred to as DN units or regressors in what follows). That is, we modeled the responses of each cell to drifting gratings and plaids as a linear combination of the responses of DN units to these same stimuli (see Supplementary Text). As regressors in this linear modeling scheme, we used either the subpopulation of DN component units or the subpopulation of DN pattern units. This allowed assessing the extent to which the tuning of a cortical neuronal subpopulation (i.e., either the pool of component or the pool of pattern cells) was captured by the tuning of the matching (or unmatching) subpopulation of units in the network.

**Fig. 8.**
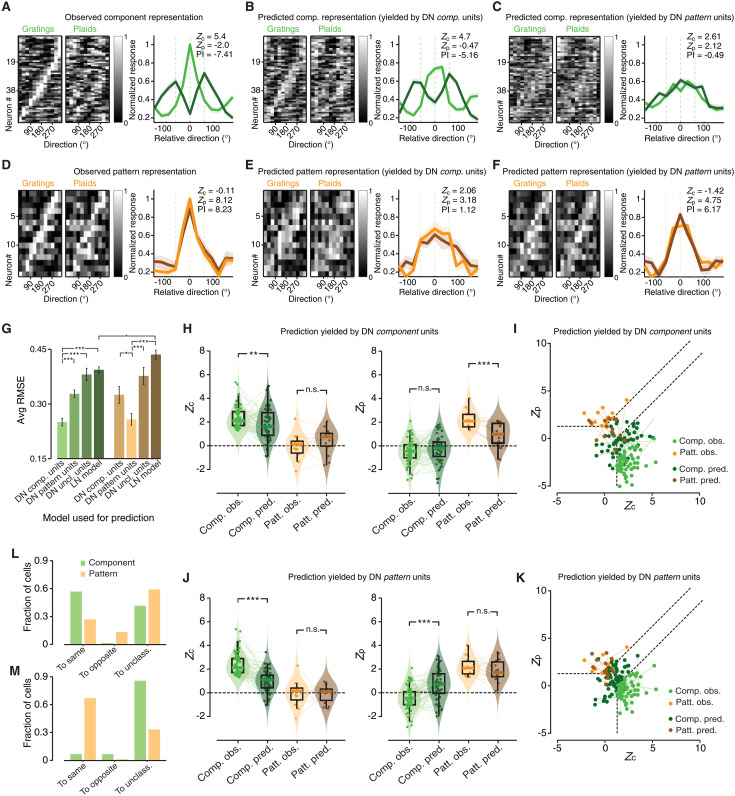
DorsalNet component and pattern units account for the tuning of matching populations of rat visual cortical neurons. (**A**) Left: Normalized responses of rat component cells, as a function of grating (left matrix) and plaid (right matrix) direction (the cells have been ordered based on the direction of their preferred grating). Right: Population averages of the direction tuning curves for gratings (light green) and plaids (dark green), with the resulting *Z*_c_, *Z*_p_, and PI indexes. Before averaging, each curve has been realigned to center the direction of the preferred grating of the cell on zero. (**B** and **C**) Same as in (A), but for the predicted (rather than measured) responses of rat component cells, as obtained by linear models using the activations of DN component (B) and pattern (C) units as regressors. (**D**) Same as in (A), but for the measured direction tuning curves of rat pattern cells. (**E** and **F**) Same as in (B) and (C), but for the predicted responses of rat pattern cells. (**G**) Bar plot displaying the error (cross-validated, population-averaged RMSE) in predicting the tuning for grating and plaid direction for the populations of component (green) and pattern (orange) cells, using the activations of DN component, pattern, and unclassified units, as well as the STA-based LN model. Error bars indicate the SE. Statistical comparisons between average RMSEs were performed using an unpaired *t* tests (*P < 0.05, **P < 0.01, and ***P < 0.001). (**H** and **I**) Same analyses as in Fig. 5 (C and D) and Fig. 5A, but for the predictions obtained by using the activations of DN component units. (**J** and **K**) Same as in (H) and (I), but with the predicted *Z*_c_ and *Z*_p_ indexes obtained by using DN pattern units as regressors. (**L** and **M**) Same analysis as in Fig. 5B, but for the predictions yielded by DN component (I) and pattern (K) regressors, respectively.

The rows of the matrix shown in Fig. 8A report the direction tuning curves of all the visual cortical neurons that were classified as component cells (i.e., same units shown in green in Fig. 5A), when probed with gratings (left) and plaids (right). The magnitude of the neuronal response is coded by the intensity of the gray scale, and the curves have been ordered based on the position of their peak response to the gratings. In the matrix on the left, the resulting diagonal band of bright pixels allows appreciating how the peak sensitivities of the component cells quite homogeneously spanned the spectrum of possible grating directions. Consistently with the nature of a component representation, the matrix reporting the tuning for the plaids featured instead two bright diagonal bands at ±60° from the band observed with the gratings. This ±60° shift of direction preference of the component cells, when tested with plaids (dark green) rather than with gratings (light green), was even more evident in the population-averaged tuning curves reported on the right (the average was performed after aligning the peaks of the normalized tuning curves for the gratings). The large magnitude of the *Z*_c_ and *Z*_p_ indexes (positive and negative, respectively) extracted from these average curves, as well as the large negative value of the PI nicely captured the component nature of the representation.

Figure 8 (B and C) reports the tuning curves of the component cells as predicted by linear models using, as regressors, the activations of the component and the pattern DN units, respectively. The component regressors accounted very well for the selectivity of the component representation, at the level of both individual and population-averaged tuning curves (compare Fig. 8B to Fig. 8A). As a result, the *Z*_c_, *Z*_p_, and PI indexes obtained from the predicted average curves matched very well the sign and magnitude of those extracted from the measured ones. By contrast, DN pattern units completely failed to capture the tuning of component cells (Fig. 8C). This can be easily appreciated by focusing on the predicted population-averaged curves, which did not match the sharpness of the measured curves for grating direction (compare the light green lines in Fig. 8, A and C). The predicted curves for plaid direction fully failed to reproduce the ±60° shift of the peak, as observed in the measured tuning curves (compare the dark green lines in Fig. 8, A and C). Not surprisingly, the *Z*_c_, *Z*_p_, and PI indexes obtained from the predicted average curves were fully inconsistent with those expected for component cells.

We found the opposite scenario when we tried to predict the tuning of pattern cells with the activations of DN component and pattern units. Figure 8D shows the tuning curves of all the cortical neurons that were classified as pattern cells (i.e., same units shown in orange in Fig. 5A), again ordered based on the direction of their preferred grating. The resulting diagonal band of bright pixels shows how the direction preference of pattern cells homogeneously spanned the whole range of grating directions (matrix on the left). As expected for a pattern representation, this band was almost fully preserved when the tuning was assessed with the plaid stimuli (matrix on the right). Such a very high consistency of the tuning measured with grating (light orange) and plaids (dark orange) can be further appreciated by comparing the population-averaged tuning curves reported on the right. The two curves were virtually identical, with an equally sharp peak centered on the same direction, resulting in a very positive *Z*_p_ index, a close-to-zero *Z*_c_ index, and a very large and positive PI (as expected for an ideal pattern cell).

Figure 8 (E and F) shows the tuning curves of the pattern cells as predicted by the activations of DN component and pattern units, respectively. The component regressors failed to account for the sharp tuning of the pattern cells for both grating and pattern direction, as shown by the broadening of the bright regions within the matrixes reporting the predicted tuning (Fig. 8E). This can be further appreciated by looking at the predicted population-averaged curves for gratings (light orange) and plaids (dark orange), whose peaks were flattened out, compared to those of the measured curves (see Fig. 8D), becoming plateaus that encompassed the whole [−60°, 60°] direction range. This is consistent with the attempt of the model to simultaneously fit the grating and pattern tuning curves, despite the component nature of the regressors. This likely forced the model to rely on those DN units with peak activations at the preferred direction of a pattern cell (to fit its grating preference), but also at ±60° from its preferred direction (to fit its plaid preference). Not surprisingly, the *Z*_p_ and *Z*_c_ indexes derived from the average curves were small and very similar, yielding a modest PI—inconsistent with that expected for a pattern cell (compare to Fig. 8D). By contrast, when DN pattern units were used as regressors, the model captured very well the tuning of pattern cells (Fig. 8F). The bright bands showing the predicted selectivity of the cells for gratings and plaids were sharp, very consistent with each other and very similar to those observed for the measured selectivity (compare to Fig. 8D). The peaks of the predicted population-averaged tuning curves were sharp and consistent for grating (light orange) and plaid (dark orange) responses, and, once more, were very similar to those of the measured average curves (see Fig. 8D). This resulted in a large positive *Z*_p_ index, a small negative *Z*_c_ index, and a very large positive PI—as expected for a pattern cell.

The trends described above were statistically assessed in Fig. 8 (G to K). In Fig. 8G, we quantified the goodness of the fit between measured and predicted tuning curves using a root mean square error metric (RMSE; see Supplementary Text) and comparing the performance of three different models: (i) linear regression with DN component units, (ii) linear regression with DN pattern units, (iii) linear regression with DN unclassified units, and (iv) the STA-based LN model. A clear ranking in terms of prediction accuracy was observed among these models. For the component cells (green bars), the best prediction (i.e., the lowest RMSE) was achieved by the model using DN component regressors (first bar). The error was significantly larger for the model using DN pattern regressors (second bar), and still larger for the model using DN unclassified regressors and for the LN model (third and fourth bars). Conversely, for the pattern cells (orange bars), the best fit was achieved by the model using DN pattern regressors (second bar), with component regressors yielding a larger error (first bar) and the LN model providing the worst performance (not surprisingly, worse also than the LN model performance in predicting the tuning of component cells).

Figure 8 (H and J) shows how consistent the *Z*_c_ (left plots) and *Z*_p_ (right plots) indexes derived from the DorsalNet predictions were with those obtained from the measured tuning curves for the component (in green) and pattern (in orange) populations (this analysis is equivalent to the one shown in Fig. 5 for the LN model predictions). Briefly, when DN component units were used as regressors (Fig. 8H), the predicted *Z*_c_ and *Z*_p_ indexes of the component population remained close to the measured ones (dark versus light green dots). Conversely, for the pattern population, the predicted *Z*_p_ index became lower than the measured one (dark versus light orange dots), consistently with the failure of this model to capture the tuning for plaid direction (as shown in Fig. 8E). As a result, while ~60% of the component cells were correctly predicted as such by the model (see Fig. 8I, dark green dots, and Fig. 8L, first green bar), less than 30% of pattern cells retained their classification (dark orange dots and first orange bar in Fig. 8, I and L, respectively). The opposite result was found when the DN pattern units were used as regressors (Fig. 8J). For the population of pattern cells, the predicted *Z*_c_ and *Z*_p_ indexes remained virtually unchanged, compared to the measured ones (dark versus light orange dots), while both indexes changed considerably for the population of component cells (dark versus light green dots). As a result, almost 70% of the pattern cells were correctly predicted as such (see Fig. 8K, dark orange dots, and Fig. 8O, first orange bar) and those that did not were nevertheless very close to the boundary between the pattern and unclassified region in the *Z*_c_/*Z*_p_ plane (Fig. 8K). On the contrary, virtually all component cells were predicted as unclassified by the model (dark green dots and third green bar in Fig. 8, K and M, respectively).

In summary, the analyses shown in Fig. 8 illustrate how the tuning of rat component and pattern cells was well captured by a linear model that used as regressors the activations of DorsalNet units, but only if the tuning of the regressors matched the tuning of the cell population to fit—DN component units only succeeded at predicting the tuning of rat component cells, while DN pattern units only properly accounted for the tuning of rat pattern cells. Overall, this further reinforces the conclusion that the pattern cells found in rat visual cortex have the functional properties of true, global motion encoders.

### The tuning of rat unclassified cells is poorly accounted for by the activations of DorsalNet units

The DorsalNet model also allowed assessing the functional properties of the cells that could be classified neither as component nor as pattern. Such unclassified neurons are way more prominent in rat (and mouse) visual cortex than in monkey dorsal stream areas (*4*–*11*), but their incidence was relatively high also in DorsalNet (see Fig. 7D). A possible scenario is that, in rodents, differently from primates, there is a continuum of tuning functions within the *Z*_c_/*Z*_p_ plane rather than a clear division into the pattern and component categories. In this scenario, pattern cells would occupy one extreme of such continuum. While this would not change the validity of our conclusions regarding their functional properties (all our analyses, including the modeling with DorsalNet, unequivocally show that rat pattern cells behave as truly nonlinear, global motion detectors), it may indicate that different (e.g., less hierarchical) mechanisms give rise to pattern cells in rodents, as compared to monkeys.

**Fig. 9.**
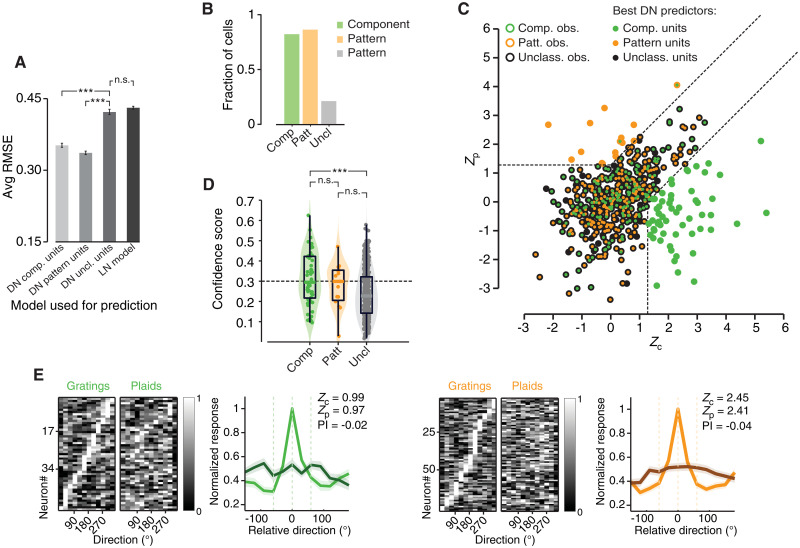
Unclassified cells behave as a qualitatively distinct population from pattern and component cells, when investigated through DorsalNet modeling. (**A**) Bar plot displaying the error in predicting the tuning for grating and plaid direction for the population of unclassified cells [same four models and statistical tests as in (G)]. (**B**) Fraction of component (green), pattern (orange), and unclassified (gray) cells for which the best DorsalNet prediction (i.e., lowest RMSE) was obtained by the matching population of DN units. (**C**) Scatter plot showing the distributions of the pairs of observed *Z*_p_ and *Z*_c_ indexes for the component, pattern, and unclassified cells (same data of Fig. 3, A to C, but pooled across areas). The outer color of each dot shows the original classification, based on the *Z*_p_ and *Z*_c_ values (green: component; orange: pattern; black: unclassified). The inner color reports which population of DN units better predicted the tonging of the cell (green: component DN units; orange: pattern DN units; black: unclassified DN units). (**D**) Confidence of the best-fitting DorsalNet models in predicting the tuning of rat cortical neurons. Confidence scores (see Supplementary Text for a definition) are plotted separately for the three classes of component (green), pattern (orange), and unclassified (gray) cells (which population of DN units best fitted the tuning is not indicated in this plot). Statistical differences were assessed using an unpaired Wilcoxon tests (***P < 0.001). (**E**) Same as in Fig. 8A, but for the two populations of unclassified cells for which DN component units (left plots) and DN pattern units (right plots) yielded the most accurate prediction of their direction tuning curves (i.e., predictions with confidence score > 0.3). These two populations are referred to as “quasi-pattern” or “quasi-component” in the main text.

To better explore the nature of unclassified cells, we compared the performance of the three different DorsalNet models described in the previous section at capturing their tuning. The model based on the activations of DN unclassified units performed poorly not only at predicting the responses of rat component and pattern cells (as previously shown in Fig. 8G) but also at explaining the tuning of rat unclassified cells (Fig. 9A, third bar). The latter was marginally better accounted for by the models based on either component or pattern DN units (first and second bars), although the RMSE was quite large (compare it to the RMSE of the best models in Fig. 8G). In more quantitative terms, while for about 80% of rat component and pattern cells, the best fit was achieved by the matching population of DN units (i.e., by the pool of component and pattern units, respectively), only for 10% of rat unclassified units the best fit was obtained with the pool of DN unclassified units (Fig. 9B).

While this result seems consistent with the continuum scenario described above, when we color-coded the best-fitting model for each rat neuron within the *Z*_c_/*Z*_p_ plane, we could not observe any clear gradient—the unclassified cells that were better predicted by DN component units were highly intermingled with those that were better predicted by the pattern units (Fig. 9C). To better understand the extent to which DN component and pattern units were effective predictors of the unclassified responses, we quantified the confidence of the prediction of the best-fitting model by computing its relative advantage (in terms of lower RMSE) compared to the other models (see Supplementary Text). The median confidence of the prediction was lower for the population of unclassified cells, as compared to component and pattern cells, although for the latter the difference did not reach significance (Fig. 9D; unclassified versus component: *P* = 4.7 × 10^−6^; unclassified versus pattern: *P* = 0.1595, unpaired *t* test). This suggests that the unclassified cells that were better modeled by DN component and pattern units were far from being proper component or pattern cells.

To further verify this, we selected two subpopulations of unclassified cells for which the model predictions yielded by DN component and pattern units had high confidence (>0.3). We plotted the tuning curves of these pools of quasi-component and quasi-pattern cells using the same layout of Fig. 8 (A to F). That is, we ordered the curves based on the position of their peaks along the grating direction axis and we computed their population averages (after aligning the peaks of the grating responses). By construction, this procedure yielded a bright diagonal band on the matrix reporting the grating responses, as well as sharp peaks (light green and orange lines) in the population average curves (Fig. 9E). By contrast, the responses to the plaid stimuli did not show any consistency with those expected for a component and a pattern representation (compare to Fig. 8, A and D). Their peaks were scattered all over the direction axis, giving rise to very flat, unstructured average tuning curves (dark green and orange lines). In summary, the tuning of these quasi-component and quasi-pattern cells had nothing to do with the one of proper component and pattern cells, as shown by small and very similar values of the *Z*_c_ and *Z*_p_ indexes, resulting in close-to-zero PIs.

In conclusion, our analysis show that rat unclassified cells have a tuning that is not properly accounted by any model we tested—not by DN unclassified regressors, likely because of the heterogeneity of both the cortical and artificial unclassified populations in terms of their selectivity for grating and plaid direction, nor by DN component and pattern regressors, which did marginally better, likely because of their regular tuning curves, but failed to identify unclassified cells with a proper component- or pattern-like behavior (see Fig. 9E). The implication of this result is further examined in Discussion.

## DISCUSSION

### Incidence of pattern and component cells in rodent visual cortex

The first goal of our study was to measure the incidence of pattern and component cells in some visual cortical areas of the rat that, by analogy with previous mouse studies (*12*–*14*), are expected to be involved in dorsal stream processing: V1, LM, and RL. Our results (Fig. 3, A to C) are in agreement with two mouse studies showing a sizable fraction of neurons that are sensitive to global motion direction in V1 (*12*, *13*), while they are at odd with another study reporting no pattern cells in this area (*14*). At the same time, they support the conclusion of the latter study that mouse LM is rich in pattern cells, but are in disagreement with the finding of an abundance of pattern cells in RL (*14*).

One possible explanation for such discrepancies is the different kind and dosage of anesthetic used during the recordings. It is well established that pattern cells are almost absent from V1 of anesthetized monkeys (*4*, *6*, *9*, *10*), while Guo *et al.* (*40*) reported a sizable amount of this cell class (9%) in awake macaques. Such responses were likely enabled by feedback from higher-level areas that are cut off during anesthesia. This suggests that, in principle, the level and kind of anesthesia can affect motion integration computations in V1. Consistently, Palagina *et al*. (*13*), who did observe pattern cells in V1, used lower isoflurane concentrations (0.6%) than Juavinett and Callaway (*14*) (from 0.6 to 1.2%), who did not. Muir *et al.* (*12*), who used a fentanyl and medetomidin anesthesia similar to the one used in our study, reported proportions of pattern and component cells in V1 (3% and 31%, respectively) that are very close to those found in our experiments (6% and 27%; Fig. 3E).

While these considerations could reconcile the contrasting results concerning the presence of pattern cells in V1 of rodents, the disagreement between (*14*) and our study on the incidence of pattern cells in RL could underlie possible differences on the functional role of this area between rats and mice. At the same time, it is worth pointing out that our results are consistent with a recent mouse study, where wide-field calcium imaging was used to investigate responses to coherent motion stimuli across the dorsal cortex of head-fixed mice (*41*). The authors found that, on average, responses to coherent motion stimuli (i.e., random dot kinematograms) are strong in the extrastriate areas that are located medially to V1 (i.e., AM and posterior medial area, or PM), as well as laterally to V1, in anterolateral area (AL), and in the most anterior and lateral part of LM. On the other hand, they reported a clear lack of strong coherent motion responses in RL, which suggests that this area may not be specialized for the processing of pure visual motion or optic flow information (differently, e.g., from monkey MT). Together with recent evidence showing that RL neurons are particularly attuned to near-field visual stimuli (*42*), as well as with the privileged position of this area, as a part of posterior parietal cortex (PPC) (*43*), to act as a hub of visuotactile integration and multimodal decision making (*44*–*47*), our results support the hypothesis that RL plays a functional role that is different from pure, high-level processing of visual motion information. On the other hand, the existence of pattern cells in such a low-level area as V1 corroborates the notion that, in rodents, primary visual cortex might contribute to motion-related computations that, in monkeys, are carried out by higher-level areas like MT (possibly reflecting the shallower hierarchy of the visual system of mice and rats). This is consistent with a recent study (*48*) reporting the presence of V1 neurons that are selective for motion streak (i.e., the smeared representations of fast-moving stimuli arising from temporal integration)—another mid-level computation of motion information that, in monkeys, is performed by MT neurons (*49*).

On the methodological front, it should be noted that our stimuli had not been independently optimized for each individual cell during recordings. Differently from monkey experiments, which are typically based on single-electrode recordings, in rodents, the use of multi-electrode arrays or optical imaging makes it highly impractical to fine-tune the SF and TF of parametric stimuli to the preference of each recorded unit. Similarly, it makes it virtually impossible to independently restrict stimulus presentation to the classical RF of each sampled neuron. Therefore, following a well-established approach in rodent visual studies, our experiments probed all units with full-field stimuli spanning a 2 × 2 combination of fixed SFs and TFs. One important question is whether this experimental choice may have biased in some way our results. We do not believe this to be the case for a number of reasons.

First, probing neurons with the same stimulus conditions allows a more unbiased exploration of neuronal tuning, under a way more naturalistic setting (natural visual scenes are not a patchwork of cell-optimized visual patterns), as compared to single-unit, cell-tailored recordings.

Second, we computed the *Z*_c_ and *Z*_p_ indexes using, for each cell, the combination of SF and TF that yielded the strongest response at the preferred grating orientation. That is, we did optimize the stimulus parameters, although within our limited range of tested TFs and SFs, before computing the metrics for the classification of the units as either pattern or component. This assured that, for each unit, the *Z*_c_ and *Z*_p_ indexes were computed using reliable, visually driven responses, resulting in clearly stimulus-modulated tuning curves, as shown by the examples of Figs. 2 and 6. More quantitatively, we verified that, for the population of recorded neurons, the SF of the RF structure, as inferred via STA (see Supplementary Text), fell squarely within the SF range of our grating/plaid stimuli (fig. S1A; see also examples in fig. S1B). For most units, the SF of the STA images ranged between 0.01 and 0.03 cpd, with the median being close to 0.02 cpd—i.e., one of the two SFs used for our drifting stimuli. This means that virtually all units reported in our study were tested with gratings and plaids presented at an SF that was close to the optimal one.

Regarding the use of full-field stimuli, an additional concern is that extraclassical RF interactions may have affected the measurement of the tuning of the recorded units. As shown in Fig. 3F and discussed in depth in the next section, we did find evidence of cross-orientation suppression, affecting similarly component, pattern, and unclassified cells. We cannot exclude that other forms of extraclassical response modulation, such as surround suppression, may also have affected our recordings. All these processes can act as powerful gain-control mechanisms. However, while they can modulate the sharpness of neuronal tuning, they do not typically alter the stimulus preference of a neuron—they can damp the peak of a tuning curve, but leaving its position unchanged (*50*, *51*). As such, it is very unlikely that these extraclassical effects played a role in determining the consistency between the tuning curves measured with gratings and plaids. Especially because the *Z*_c_ and *Z*_p_ metrics used to assess such consistency are based on computing Pearson correlation coefficients, which are, by definition, scale invariant. That is, the *Z*_c_ and *Z*_p_ metrics are strongly sensitive to the relative position of the peaks along the tuning curves, but not to their magnitude. To our knowledge, the only way by which surround modulation mechanisms may shift the positions of peaks along a tuning curve is when the surround stimulus is appositely designed to only interfere with one of the constituent features of the central stimulus. For instance, Trott and Born (*52*) found that the peak of the tuning curve for static plaids presented within the classic RF of monkey V1 neurons shifted when the surround stimulus was a grating matching the orientation of one of the component gratings of the plaid. Obviously, this scenario does not apply to our experiments, where the visual pattern impinging upon the classical RF of the recorded units was the same as the one shown in the surround, both in the case of the grating and of the plaid stimuli—thus assuring, in the latter case, that any suppressive effect of the surround affected equally strongly both component gratings.

An additional concern regarding the use of full-field stimuli presented in a rectangular aperture (i.e., the stimulus display), rather than through a circular window, as typical of monkey studies, is the possibility that the neurons’ RFs overlapped the edges of the display. The intersections between these edges and the contours of the gratings could provide unambiguous (although not veridical) pattern direction clues (known as “terminators”), as in the well-known barber-pole illusion, where gratings drifting diagonally are perceived as drifting along the longer edge of a rectangular aperture (*53*). In the monkey, late responses of MT neurons to drifting gratings viewed through elongated rectangular apertures have been shown to depend on the direction of motion of the terminators along the long axis of the aperture (*54*). This effect, however, requires the terminators to move within the classical RF of the neurons, i.e., it requires most of the rectangular aperture to be contained within the neuronal RF. Again, this scenario does not apply to our experiments, where the neuronal RFs were much smaller than the stimulus display/aperture, thus being contained within it, rather than vice versa (see examples in Fig. 2). Still, we checked how many cells had RFs that intersected the monitor’s edges, by plotting the RF boundaries, as inferred by the STA images, within the stimulus display. Virtually all components and pattern cells in the three visual areas had RFs located near the center of the display, and their boundaries did not cross or include the edges of the display (fig. S1, C and D). This concentration of RFs at the center of the display was also visible when looking at the population averages of the RFs for the two cell classes in the three areas (fig. S1E). Therefore, we can confidently rule out that sensitivity to terminators played a role in determining the classification of pattern and component cells as such.

### Spatial integration properties of rat pattern and component cells

The second goal of our study was to characterize some key spatial integration properties of rat pattern and component cells. We started by assessing the extent to which cross-orientation suppression affects component and pattern cells in rat visual cortex. Addressing this question is important for two reasons. First, cross-orientation suppression belongs to a class of nonlinear tuning phenomena that are thought to depend on divisive normalization, a canonical cortical computation where the response of a neuron is divided (normalized) by the summed activity of a pool of nearby neurons. Divisive normalization has been called into cause to explain a variety of nonlinear interactions among competing stimuli within the classical and extraclassical RFs of visual neurons in cats and monkeys (*51*). There is now a strong interest in establishing whether the same interactions take place in rodent visual cortex, because of the potential of dissecting the underlying neuronal circuitry using the molecular tools that rodent studies allow (*50*).

This result is also important because it allows ruling out one of the mechanisms we have previously hypothesized to account for the observed asymmetry in the ability of rats to generalize a motion discrimination task from grating to plaids or vice versa. For a thorough discussion of this topic, we refer the reader to our previous study (*16*). Here, we can only briefly mention that, since responses to plaids were generally weaker than responses to gratings for both pattern and component cells (Fig. 3E), our previous modeling work suggests that component cells act as the building blocks that are necessary to “assemble” pattern cells, but only the latter are read out by decision neurons to infer motion direction. As explained in (*16*), this is a scenario where rat pattern cells, despite their scant number and their presence in multiple visual areas, sit higher than component cells along the functional motion processing hierarchy, thus performing a similar role to that of monkey MT neurons (*9*). This conclusion is consistent with the well-established ability of rodent visual cortical neurons to send (receive) functionally specific inputs to (from) downstream (upstream) areas (*55*–*59*). Projection-specific or axonal imaging experiments targeted at functionally characterizing the input from V1 and LM to higher-order areas involved in perceptual decision making such as ACC, M2 (*60*), or PPC (*44*, *45*, *61*) could test directly this hypothesis, by verifying whether the populations of projecting neurons relaying visual information to these areas are particularly enriched in pattern cells.

Another spatial integration property that we assessed was the phase invariance of pattern and component responses. Large phase invariance results in sustained, poorly modulated responses to the presentation of drifting gratings and distinguishes complex cells from the phase-sensitive (or position-sensitive) simple cells. To our knowledge, the relationship between pattern/component and complex/simple cells has not been systematically explored in the monkey literature. On the basis of computational considerations (*8*, *32*, *33*), the two classes of nonlinear units, i.e., pattern cells and complex cells, should pool over different set of afferents, consistently with their distinct functional role in, respectively, dorsal and ventral stream processing—i.e., to provide a pattern-invariant encoding of motion direction, in the case of pattern cells, and to provide a translation-invariant encoding of orientation, in the case of complex cells. This would suggest that pattern- and complex-like tuning emerges independently across visual cortex, with some units displaying both kinds of nonlinearities (i.e., pattern cells with complex-like spatial tuning) and other units displaying only one kind (i.e., simple cells with pattern-like selectivity or component cells with complex-like tuning). Conversely, since the ventral and dorsal stream shares the initial processing stages (V1 and V2 in primates), this could bring to a hierarchical increase of both kind of nonlinearities, with the pattern/complex combination being more likely than the pattern/simple or component/complex combinations.

Our data are consistent with the notion that pattern- and complex-like tuning develops independently, since several units could be found with pattern/simple or component/complex tuning (Fig. 6A). At the same time, we also observed a tendency for pattern cells to have significantly lower phase sensitivity (i.e., lower MI) than component cells—i.e., to be more consistent with a complex-like behavior (Fig. 6B). This tendency is by no means as strong as the one observed in deep regions of the rat ventral stream, such as area LL, where the median MI has been measured to be 1.28 (*25*). Still, the slight propensity of pattern cells to be more phase invariant than component cells is consistent with the equivalence between rodent LM (where we found the largest proposition of pattern cells) and monkey V2 that has been proposed by several neurophysiological and neuroanatomical studies (*14*, *62*–*65*) and, therefore, with the role of the former as a gateway to both dorsal and ventral processing in rodents.

In anticipation of what discussed in the next section, it is worth mentioning that a much weaker co-occurrence of pattern- and complex-like tuning was found for the units of DorsalNet, a deep neuronal network model of the dorsal stream (fig. S2). This is not surprising, since that network was designed to solve a typical dorsal-processing task and, as such, lacked a specific module devoted to ventral processing. The fact that DorsalNet units nevertheless displayed a tendency to become more complex-like in deep layers suggests that acquiring some degree of translation invariance is useful for motion-related computations. At the same time, the fact that phase invariance increase of virtually the same amount for pattern and component units indicates, once again, that the computations underlying pattern-invariant encoding of motion direction and translation-invariant encoding of orientation are independent.

### Reverse correlation versus DorsalNet as models of rat component and pattern cells

The last and more important goal of our study was to assess whether pattern cells in rodents perform a truly integrative, nonlinear processing of local motion signals to encode global motion direction of complex patterns, as the plaid stimuli. Answering this question is important because, as originally pointed out by (*17*) and shown by the example we provided in Fig. 1, tuning consistent with pattern-like behavior may emerge from blobby, purely linear, Gabor-like RFs with low aspect ratio, as those expected to be found in rodents (*23*, *25*).

To address this issue, we applied reverse correlation analysis (STA) to reconstruct the linear RF of units classified as either component or pattern cells by processing their responses to spatiotemporally correlated noise movies (see Materials and Methods and Supplementary Text for details). As expected, for many component cells, we obtained temporal sequences of high-contrast STA images (Fig. 4, A and B), typically Gabor-like (Fig. 4C), often containing a pair of flanking lobes with opposite polarity (i.e., one excitatory and the other inhibitory; Figs. 2 and 4, A and D). In general, these STA images, when used as the linear kernels in an LN model, predicted the tuning of component cells for gratings and plaids well enough to keep the resulting *Z*_p_ and *Z*_c_ index values close to those observed empirically (Fig. 5A). As a result, most component cells retained their classification when the *Z*_p_ and *Z*_c_ indexes were computed on the responses predicted by the model (Fig. 5B). Also, pattern cells yielded in many cases well-structured STA images, with a quality that, in general, was only slightly lower than that of the STA images obtained for component cells (Fig. 4). However, the ability of STA-based NL models to account for direction tuning was strikingly poorer for pattern cells—none of the neurons classified as pattern on the ground of their observed responses retained its classification when the *Z*_p_ and *Z*_c_ indexes were computed on the responses predicted by the model (Fig. 5, A and B). This suggests that, although STA can capture some linear “residue” of the spatiotemporal selectivity of pattern cells, it is not able to account for their tuning. This rules out the possibility that the pattern responses we observed (e.g., see Fig. 2, D to F) are the result of linear filtering processes performed by blobby RFs (of the kind illustrated in Fig. 1C). On the contrary, our results suggest that the computations carried out by pattern cells to encode motion direction rely on nonlinear processes that STA (by construction, being a linear method) cannot capture.

This conclusion has important implications for our understanding of the nature of motion processing in rodent visual cortex. Looking at the primate dorsal stream literature, the most established mechanistic models of pattern-motion selectivity in MT are based on the nonlinear pooling of inputs from narrowly tuned, V1-like component cells, whose preferred motion directions are spread over a wide range of angles (*8*). We still do not know the extent to which such models can be extended to rodents. Certainly, they will need to be adapted to fit the specificities of the rodent visual system—e.g., to account for the larger contribution of direction-selective inputs from retinal ganglion cells (*66*) and the overall broader tuning of Lateral Geniculate Nucleus (LGN) and intracortical inputs (*13*, *23*, *25*, *55*, *67*). Our study, however, suggests that, also in rodents, the tuning of pattern cells is mainly determined by truly nonlinear, integrative processes, possibly homologous to the ones at work in primates and instantiated in the abovementioned models.

To test further this hypothesis, it would be interesting to compare our findings to similar analyses performed on monkey pattern cells. Unfortunately, we are not aware of monkey studies in which STA was applied to characterize the structure of component and pattern cells and then to predict their direction tuning, as done here. To our knowledge, the only reverse correlation study in which the RF structure of MT neurons was mapped using very sparse noise did not differentiate between component and pattern cells, and neither measured nor predicted neuronal responses to gratings and plaids (*68*).

This is one of the reasons we performed a comparison with a state-of-the-art neural network model of the dorsal stream: DorsalNet (*26*). One of our goals was to compare the tuning properties of component and pattern cells recorded from rat visual cortical areas to those of the units of a benchmark computational architecture, where selectivity for increasingly complex motion patterns is built via hierarchical processing. As shown by (*26*), the selectivity of neurons sampled from progressively higher stages of the monkey dorsal stream is better explained by the activations of units located at progressively deeper layers of DorsalNet—i.e., layers 1, 2, and 3 best match areas V1, MT, and MST. Our analysis of the tuning of DN units for gratings and plaids corroborated this conclusion, showing that, while the proportion of component units decreases along the hierarchy, the fraction of pattern units increases (Fig. 7D)—the same trend found along the monkey dorsal stream (*9*). Together, these findings indicate that DorsalNet successfully captures some of the core hierarchical processes underlying motion representation along the monkey dorsal stream. This makes DorsalNet extremely valuable as a benchmark against which to compare the properties of a system, such as rodent visual cortex, whose level of sophistication in terms of motion processing is still poorly understood, at least as compared to primate visual cortex.

Our analyses show that three key phenomena we observed in the rat visual cortex also occur in DorsalNet: (i) the presence of both component and pattern units, with an increase in abundance of the latter in higher stages of processing, possibly reflecting what we observed between V1 and LM (compare Fig. 7D to Fig. 3E); (ii) the similar impact of cross-orientation suppression on these two classes of units (compare Fig. 7E to Fig. 3F); and (iii) the success of linear spatiotemporal filters inferred via STA to account for the tuning (and, therefore, the classification) of component units but not of pattern units (compare Fig. 7, H to K, to Fig. 5). The latter result is particularly important. Finding the same evidence for integrative, nonlinear computations being performed by rat pattern cells and DN pattern units further corroborates a potential homology with the monkey dorsal stream.

To further strengthen this conclusion, we also modeled (via linear regression) the tuning of rat component and pattern cells for grating and plaid direction using the activations of component and pattern DN units. DN component units successfully captured the tuning of rat component cells, but not of pattern cells. Vice versa, DN pattern units accounted for the tuning of pattern cells, but not of component cells. This confirms that the functional properties of rat pattern cells can only be captured by models that are capable to encode global motion direction (like the pool of DN pattern units)—models encoding local direction only (like the STA-based LN models or the set of DN component units) are not up to the task. At the same time, our results show that the reverse is also true—global motion encoders do not succeed at modeling the selectivity of local motion encoders, as rat component cells. This may explain why in multiple species (monkeys, rats, and mice), as well as in DorsalNet, populations of pattern and component cells coexist at every processing stage (although in very different proportions, depending on the system). Maintaining the ability to extract the direction of the elemental, constituent features (the components) of global patterns could be of some functional relevance in the processing of motion information. If the hierarchical buildup of motion integration would ultimately result in a population of pattern cells only, sensitivity to the local direction of the component features would be fully lost.

We also tried to predict the tuning of rat unclassified cells, using various subpopulations of DN regressors, but neither the unclassified units nor the component or pattern units yielded good fits (Fig. 9). Our modeling allowed establishing that the unclassified pool did not contain subsets of neurons with a tuning close to that of component and pattern cells (Fig. 9E). This indicates that rat pattern and component cells form two categories that are clearly distinct from the larger population of unclassified neurons. This makes it unlikely that their tuning, especially in the case of pattern cells, simply emerges as the extreme tail of a very broad spectrum of selectivity profiles over the *Z*_c_/*Z*_p_ plane. Rather, the class of pattern cells seems to be the result of dedicated nonlinear integrative mechanisms, possibly not dissimilar from those at work in the monkey dorsal stream.

In summary, our experimental findings, supported by the comparison with the tuning properties of DN units, provide compelling evidence for the existence of truly advanced encoders of global motion direction distributed across rat visual cortical areas V1 and LM. As such, our study sets the stage to exploit the molecular and genetic tools that are available in rodents to dissect the circuit-level mechanisms underlying integration of local motion signals [e.g., see (*69*)] into the representation of global motion direction.

## MATERIALS AND METHODS

### Surgery and recordings

All animal procedures were approved by the Institutional Animal Care and Use Committee of the International School for Advanced Studies (SISSA) and by the Italian Ministry of Health (project DGSAF 22791-A, submitted on 7 September 2015 and approved on 10 December 2015, approval 1254/2015-PR).

We performed extracellular neuronal recordings from 29 naïve male Long Evans rats, weighted 300 to 700 g and aged 3 to 12 months. Each rat was anesthetized with an intraperitoneal injection of a solution of 0.3 mg/kg of fentanyl (Fentanest, Pfizer) and 0.3 mg/kg of medetomidin (Domitor, Orion Pharma). During the surgery, we monitored the anesthesia level by checking the animal paw reflex and by measuring the oxygenation and heart rate through a pulse oximeter (Pulsesense-VET, Nonin). Temperature was monitored and maintained at 37°C through a heating pad, and a constant flux of oxygen was delivered to the animal throughout the surgery. A constant level of anesthesia was maintained by continuously delivering an intraperitoneal injection of the same aesthetic solution used for the induction, but at a lower concentration (0.1 mg/kg per hour of fentanyl and 0.1 g/kg per hour of medetomidin), by means of a syringe pump (NE-500; New Era Pump Systems). Once deeply anesthetized, the animal was secured to a stereotaxic apparatus (Narishige, SR-5R) and we performed a craniotomy on the left hemisphere, over the selected target (typically, a ~4 mm^2^ window). Stereotaxic coordinates of the center of the craniotomy were 6.5 mm posterior from bregma and 4 mm left to the sagittal suture [i.e., anteroposterior (AP), 6.5; mediolateral (ML), 4.0] for sessions targeting V1, 7 mm AP and 5 mm ML for sessions targeting ML, and 5 mm AP and 4.5 mm ML for sessions targeting RL.

To keep eyes hydrated during the surgery, we protected them by applying an ophthalmic ointment (Epigel, Ceva Vet). Once the surgery was completed, the rat was placed over a rotating platform (used to place the RFs of the recorded neurons on the center of the monitor), with the right eye just in front of the center of the screen (distance = 30 cm) and the left eye covered with nontransparent black tape. The right eye was fixed through a metal eye-ring to prevent eye movements during the visual stimulation protocol.

Extracellular recordings were performed under light anesthesia while rats were passively exposed to visual stimulation. Recordings were carried out using single-shank (or double-shank) 32-channel (or 64-channel) silicon probes (NeuroNexus Technologies) with a site recording area of 775 μm^2^ and an intersite spacing of 25 μm. The insertion of the electrode into the cortex was performed through an oil hydraulic micromanipulator (Narishige, MO-10). The insertion depth was different for each area: For V1 and RL, it was ~900 μm with an insertion angle relative to the cortical surface of ~20°; for LM, it was ~1500 μm, with an insertion angle of ~25°. Neuronal signals were recorded with a sampling rate of 25 kHz, and were acquired and preamplified using a system three TDT (Tucker-Davis Technologies) workstation.

Here, we did not reconstruct the cortical depth and laminar location of the recording sites. However, based on our previous work, where recordings were performed using the same experimental approach, we can speculate that most cells were recorded from layer 5 and, to a lesser extent, from layer 4 (*25*, *31*).

### Single-unit isolation

Single units were isolated offline using the KlustaKwik-Phy software package (*70*). After the automatic spike detection and features extraction, we performed a manual refinement of the sorting through the “Kwik-GUI” interface, based on the following criteria: (i) the compactness of the clusters in the space of the principal components of the waveforms, (ii) the shape of the auto- and cross-correlogram (the latter was used to decide whether to merge or not two clusters), (iii) the variation of the principal components of the waveform over time, (iv) the shape of the average waveform. To be included in the analyses presented in Results, single units were required to meet the following criteria: (i) show a clear refractory period (i.e., less than 0.5% of the spikes present in <2 ms of the spikes’ autocorrelogram) and (ii) be clearly grating or plaid responsive, i.e., with the response to the most effective grating or plaid condition being larger than two spikes per second (baseline-subtracted) and being larger than six *z*-scored points relative to baseline activity. The average baseline (spontaneous) firing rate of each well-isolated unit was computed by averaging its spiking activity over every interstimulus interval.

### Visual stimuli

During each recording session, two kinds of visual stimulation protocols were administered to the rats. The first one was an RF mapping procedure, aimed at estimating the average preferred retinotopic location of the units recorded at each site along the length of the probe. This protocol consisted in the presentation of 10° long drifting bars spanning different orientations (0°, 45°, 90°, 135°) and shown against a black background in 66 different positions, across a grid of 6 rows (spanning vertically 50°) and 11 columns (spanning horizontally of 100°). This procedure allowed identifying the visual area each unit was recorded from, by tracking the reversals of the retinotopy that, in rodents, take place at the boundaries between adjacent visual areas, as described in several other studies from our group (*25*, *65*, *71*).

The second visual stimulation protocol included all the stimuli used to characterize neurons as pattern or component cells and to reconstruct their linear RFs, as described in the main text of this paper. The protocol consisted of (i) 20 repetitions (trials) of 1.5-s-long, full-contrast drifting gratings, made of all possible combinations of two SFs (0.02 and 0.04 cpd), two TFs (2 and 6 Hz), and 12 directions (from 0° to 330°, in 30° increments); (ii) 20 repetitions of 1.5-s-long drifting plaids (made of two superimposed, half-contrast drifting gratings with a 120° cross-angle), again spanning all possible combinations of two SFs (0.02 and 0.04 cpd), two TFs (2 and 6 Hz), and 12 directions (from 0° to 330°, in 30° increments); and (iii) 20 different 60-s-long spatially and temporally correlated, as well as contrast-modulated noise movies, which were built as described in (*31*). All stimuli were randomly interleaved, with a 1-s-long interstimulus interval, during which the display was set to a uniform, middle-gray luminance level. Stimuli were generated and controlled in MATLAB (MathWorks) using the Psychophysics Toolbox package and displayed with gamma correction on a 47-inch LCD monitor (SHARP PNE471R) with 1920 × 1080–pixel resolution, a maximum brightness of 220 cd/m^2^, and spanning a visual angle of 120° azimuth and 90° elevation (placed at 30 cm from the eye of the animal). Grating stimuli were presented at 60-Hz refresh rate, whereas noise movies were played at 30 Hz.

### Tuning metrics

We quantified direction selectivity of single units in each area by computing the DSI:

$$\mathrm{DSI}=(R_{\mathrm{pref}}-R_{\mathrm{opposite}})/(R_{\mathrm{pref}}+R_{\mathrm{opposite}})$$

where *R*_pref_ is the response of the neuron to the preferred direction and *R*_opposite_ is the response to the opposite direction. The response of a neuron to a stimulus in a given direction *x* (i.e., *R_x_*) was defined as the trial-averaged firing rate, computed over the entire stimulus presentation window and *z*-scored with respect to the spontaneous activity, as computed during all interstimulus intervals (negative values were clipped to zero). Neurons with DSI > 0.33 were categorized as direction selective.

To distinguish simple from complex cells, the phase-dependent modulation of the neuronal responses at the TF *f*_1_ of a drifting grating was quantified by an MI adapted from (*72*) and used in (*25*, *31*, *34*), defined as:

$$\mathrm{MI}=|\frac{\mathrm{PS}(f_{1})-{\left\langle \mathrm{PS}\right\rangle }_{f}}{\sqrt{{\left\langle {\mathrm{PS}}^{2}\right\rangle }_{f}-{\left\langle \mathrm{PS}\right\rangle }_{f}^{2}}}|$$

where PS indicates the power spectral density of the stimulus-evoked response, i.e., of the peristimulus time histogram, and 〈 〉*_f_* denotes the average over frequencies (see Fig. 6 and fig. S2).

Classification of neurons as “pattern,” “component,” or “unclassified” was based on their *z*-scored, Fisher-transformed, partial correlation indexes (*Z*_p_ and *Z*_c_), whose definition, derived from the monkey dorsal stream literature (*4*, *27*), is reported in Supplementary Text.

### Linear RF reconstruction and prediction of pattern and component responses

Reconstruction of linear RFs underlying the selectivity of the recorded neurons was achieved using the STA technique (*28*–*30*). The method was applied to the spike trains fired by the neuron in response to the spatiotemporally correlated noise movies (see above). The STA method yields an ordered sequence of images (i.e., spatial filters), each representing the average of the stimulus ensemble at a given time lag from spike generation. STA images can therefore be interpreted as the (linear) spatiotemporal RF of a given neuron.

As detailed in Supplementary Text and in our previous studies (*25*, *31*), STA images were first decorrelated and then their statistical significance was assessed pixelwise through a permutation test yielding *z*-scored STA intensity values (as those shown in Figs. 2, 4, and 7). Finally, the reconstructed, spline-interpolated STA image sequences were used as input stage filters of a classical LN model. This yielded predicted tuning curves for gratings and plaids, as those shown in Fig. 2 and Fig. 7, A and B (dashed lines). These curves were then used to recompute the *Z*_p_ and *Z*_c_ indexes (see previous section) and (re) classify each unit as component, pattern, or unclassified (see Figs. 5 and 7, H to K).

To estimate the amount of signal contained in a given STA image, we used the CI metric that we have introduced and applied in previous studies (*25*, *31*) and is briefly described in Supplementary Text.

### Quantification of cross-orientation suppression

To quantify the amount of suppression (or enhancement) of the response of each unit to the plaid as compared to the grating stimuli, we defined a normalized CSI as:

$$\mathrm{CSI}=\frac{\left(R_{g}-R_{p}\right)}{\left(R_{g}+R_{p}\right)}$$

Here, *R*_p_ and *R*_c_ indicate the peak responses to plaids and gratings, respectively (i.e., the responses to the two stimuli when presented at the most effective directions). This index takes a value of 1 for a unit responsive to gratings but not to plaids (i.e., extreme suppression) and, vice versa, a value of −1 for a unit responsive to plaids but not to gratings (i.e., extreme enhancement), whereas it takes a value of 0 for a unit showing the same peak response firing rate for both gratings and plaids. To correctly interpret this index, attention should be paid to the fact that both gratings and plaids were presented at full contrast. This was done to elicit stronger responses and increase the yield of the recordings, as well as to match the contrast of the stimuli used in our previous behavioral study (*16*). A more rigorous assessment of cross-orientation suppression would have required matching the contrast of the constituent gratings of the plaids to the contrast of the gratings presented in isolation. However, since the purpose of our study was to compare the level of suppression between the two categories of component and pattern cells, and since both cell classes were probed with the same grating and plaid stimuli, our comparison is not affected by the fact that the latter were both displayed at the same maximal contrast. The main goal of measuring the level of cross-orientation suppression in the two cell classes was to test which of the two mechanisms of motion integration we proposed in (*16*) can better account for rat perception of plaid motion direction—hence, the need of matching the contrast of the stimuli used in the two studies.

### DorsalNet simulations

To help interpret the results of our analyses of visual neuronal responses, we decided to compare them with those obtained by applying the same analysis pipeline (Fig. 7) to a state-of-the-art computational model of dorsal processing: DorsalNet, a six-layer 3D convolutional neural network recently proposed as the best-in-class in silico model of the dorsal stream (*26*). We also used the activations of DorsalNet units to build models of direction tuning for rat component, pattern, and unclassified cells. Both approaches are described in Supplementary Text.

### Statistical analysis

All statistical analyses were computed in MATLAB (MathWorks) 2018a and 2019b using custom software (implementing STA permutation test and *z*-scoring, based on “chi2cdf” MATLAB function for χ^2^ tests) as well as using MATLAB built-in statistical functions “ttest2,” “signrank,” and “ranksum” (for *t* tests and Wilcoxon tests). The dataset includes data from 29 animals for a total of 447, 367, and 412 well-isolated single units in areas V1, LM, and RL, respectively. Out of this, 258, 187, and 184 were selected as stimulus responsive and included in the analyses presented in the study. The sample size of different neuronal subpopulations meeting specific response-property criteria that were used for each analysis is reported in the main text.
