# Supplementary material for: Truly pattern: Nonlinear integration of motion signals is required to account for the responses of pattern cells in rat visual cortex
Source: Sci Adv. 2023 Nov 8;9(45):eadh4690. doi: 10.1126/sciadv.adh4690 (PMC10631736; doi:10.1126/sciadv.adh4690)
Supplement: Supplementary file 1 — Supplementary Text Figs. S1 and S2 Legend for movie S1 [file sciadv.adh4690_sm.pdf]

Science Advances
AAAS

Supplementary Materials for

Truly pattern: Nonlinear integration of motion signals is required to account for the responses of pattern cells in rat visual cortex

Giulio Matteucci *et al.*

Corresponding author: Davide Zoccolan, zoccolan@sissa.it

Sci. Adv. **9**, eadh4690 (2023)
DOI: 10.1126/sciadv.adh4690

The PDF file includes:

Supplementary Text
Figs. S1 and S2
Legend for movie S1

Other Supplementary Material for this manuscript includes the following:

Movie S1

Supplementary Text

Classification of pattern and component responses

Classification of neurons as "pattern", "component" or "unclassified" was based on their z-scored, Fisher-transformed, partial correlation indexes (Z_p and Z_c), whose definition, derived from the monkey dorsal stream literature (*4*, *27*), is the following:

$$P_c = \frac{(r_c - r_p r_{pc})}{\sqrt{\left((1 - r_p^2)(1 - r_{pc}^2)\right)}} \qquad P_p = \frac{(r_p - r_c r_{pc})}{\sqrt{\left((1 - r_c^2)(1 - r_{pc}^2)\right)}}$$

$$Z_c = \frac{\sqrt{(n-3)}}{2} \ln \frac{1 + P_c}{1 - P_c} \qquad Z_p = \frac{\sqrt{(n-3)}}{2} \ln \frac{1 + P_p}{1 - P_p}$$

Here: i) r_c is the correlation coefficient between the observed tuning curve for the plaids and the predicted tuning curve under the assumption that the unit behaves as an ideal component cell; ii) r_p is the correlation coefficient between the observed tuning curve for the plaids and the predicted tuning curve under the assumption that the unit behaves as an ideal pattern cell; iii) r_{pc} is the correlation coefficient between the two predicted curves; and iv) n is the number of elements of the tuning curves. The ideal pattern-cell behavior is simulated by imposing that the tuning curve is the same in response to both gratings and plaids – i.e., the predicted tuning curve for the plaids is trivially obtained by setting it identical to the tuning curve obtained for the gratings. The ideal component-cell behavior is simulated by imposing that the unit, when presented with a plaid, responds to its constituent gratings in the same way as it would respond if the gratings were presented in isolation – i.e., the predicted tuning curve for the plaids is obtained by shifting both leftwards and rightwards of half plaid cross-angle the tuning curve measured for the gratings and then averaging the two shifted versions.

At 90% confidence (as usually done in the literature) the Z critical value of 1.28 defines the pattern and component regions of the (Z_c, Z_p) plane, as follows:

pattern condition:

$$Z_c - 0 > 1.28 \text{ if } Z_c < 0$$

$$Z_p - Z_c > 1.28 \text{ if } Z_c > 0$$

component condition:

$$Z_c - Z_p > 1.28 \text{ if } Z_p > 0$$

$$Z_c - 0 > 1.28 \text{ if } Z_p < 0$$

Neurons falling in these regions are classified, respectively, as pattern or components. Neurons that do not meet these criteria are considered unclassified.

Linear RF reconstruction and prediction of pattern and component responses

Reconstruction of linear RFs underlying the selectivity of the recorded neurons was achieved employing the Spike-Triggered Average (STA) technique (*28–30*). The method was applied to the spike trains fired by the neuron in response to the spatiotemporally correlated noise movies (see the Materials and Methods). The STA method yields an ordered sequence of images (i.e., spatial filters), each representing the average of the stimulus ensemble at a given time lag from spike generation. STA images can therefore be interpreted as the (linear) spatio-temporal RF of a given neuron.

As we did in previous studies (*25*, *31*), we took into account the correlation structure of our stimulus ensemble (determined by the spatiotemporal correlations in the noise movies) to reduce artefactual blurring of the reconstructed filters that such correlation could induce. To this end, we decorrelated the STA images by dividing them by the covariance matrix of the whole stimulus ensemble (*29*), using Tikhonov regularization to handle covariance matrix inversion.

Statistical significance of STA images was assessed pixelwise, by applying the following permutation test. After randomly reshuffling the spike times, the STA analysis was repeated multiple times (n = 30) to derive, for each pixel, a distribution of intensity values under the null hypothesis of no linear stimulus-spike relationship (i.e., random spikes being fired in a fully uncorrelated way with respect to the visual input). This allowed z-scoring the actual STA intensity values using the mean and standard deviation of these null distributions independently for each pixel. The temporal span of the spatiotemporal linear kernel reconstructed via STA extended from the time of spike generation up to 330 ms earlier. This corresponds to a duration of 10 frames of the noise movie played at 30 Hz. These procedures were performed on downsampled noise frames (16x32 pixels). The resulting STA images were then spline interpolated at higher resolution for ease of visualization and analysis, as well as to derive the subsequent LN predictions (see next paragraph).

To derive linear predictions for the grating and plaids tuning curves, as expected given the reconstructed STA image sequences, we used the latter as input stage filters of a classical linear-nonlinear (LN) model (*29*). Specifically, to obtain a prediction of the response to a stimulus drifting in a given direction, the sequence of frames of the stimulus movie was fed as an input to the STA-estimated linear filter. The output of the filter was then passed through a rectified linear unit (a.k.a. relu) to obtain the final response of the model to each stimulus frame. In the analysis presented in Fig. 5, this was done for the stimuli with SF = 0.02 cpd and taking the preferred TF for each neuron (i.e., 2 or 6 Hz). This choice was dictated by the need of carrying out the analysis with gratings and plaids matching at best the dominant spatial frequency of the reconstructed linear receptive fields, so as to maximize the quality of predictions. For this reason, the analysis reported in Fig. 5 was carried out on the subset of pattern and component neurons maintaining at SF = 0.02 cpd their pattern or component classification (determined, in the analysis of Fig. 3, at their preferred SF, i.e., either 0.02 or 0.04 cpd). To allow overfitting and implement a test/train split, the gain of the nonlinear activation function was determined, for each neuron, by fitting the LN model to the responses of the unit to the gratings presented at the non-preferred TF (i.e., the fit was not carried out on the responses to the stimulus conditions analyzed in Fig. 5). The fit was performed using the MATLAB “fminunc” function. When carrying out the analysis on DorsalNet units, the gain

was instead fixed to one (being this the gain of the relu functions implemented in network). Once obtained a predicted response to the drifting stimulus under consideration, this was averaged in time over the stimulus presentation window to get the final predicted response rate of the unit for that stimulus. Repeating this process for each direction of a given stimulus class (i.e., gratings of plaids) yielded predicted tuning curves, as the one shown in Fig. 2 and Fig. 6A-B (dashed lines). These curves were then used to re-compute the Z_p and Z_c indexes (see previous section) and (re)classify each unit as “component”, “pattern” or “unclassified” (see Fig. 5 and Fig. 6H-M).

Linear RF structure quantification

To estimate the amount of signal contained in a given STA image, we used the “contrast index” (CI) metric that we have introduced and applied in previous studies (*25*, *31*). Briefly, the CI is designed to be a robust measure of maximal local contrast in any STA image. Having expressed the intensity values of each STA image in terms of z-scores (against the spike-time shuffled null distributions; see previous section), the CI value is defined as the maximal local peak-to-peak (i.e., white-to-black) difference in the STA, expressed in sigma units. The local peak-to-peak distance computation was performed using MATLAB “rangefilt” function. The locality of the CI is determined by the size of the neighborhood used by rangefilt. In our analyses, this parameter was set to be 20% of the lower dimension (the height) of the reconstructed STA filter. For the analysis reported in Fig. 4, all STA images for each neuron in the component, pattern, unclassified categories were included. As in done our previous studies (*25*, *31*), we also characterized the structural complexity of the linear RFs yielded by STA by counting the number of excitatory/inhibitory lobes that were present in a STA image. The procedure is similar to the one described in (*25*). Briefly, we applied a binarization threshold over the modulus of the z-score values of the image (at 3.5 units of standard deviation). We then computed the centroids of the simply connected regions within the resulting binarized image (i.e., the candidate lobes). Last, we applied a refinement procedure, which is detailed in (*25*), to prune spurious candidate lobes (i.e., lobes smaller than 5% of the width of the reconstructed STA filter were rejected). As a final quantification of the linear RF structure of the recorded units, we fitted a Gabor function to each STA image (using the “fit2dGabor” function by Gerrit Ecke: https://www.mathworks.com/matlabcentral/fileexchange/60700-fit2dgabor-data-options) and we evaluated the goodness of this fit by computing the coefficient of determination R^2 (as reported in Fig. 4C and 7G).

DorsalNet simulations

To help interpreting the results of our analyses of visual neuronal responses, we decided to compare them with the those obtained by applying the same analysis pipeline to a state-of-the-art computational model of dorsal processing. To this aim, we choose DorsalNet, a 6-layer 3D convolutional neural network recently proposed as the best-in-class in-silico model of the dorsal stream (*26*). DorsalNet is a 3D ResNet trained with the self-supervised learning objective of predicting the parameters of simulated self-motion of an agent moving in a simulated environment from its own visual input. The training dataset consisted of a set of short videos (10 frames) of self-motion generated with the AirSim package, a drone and land vehicle simulation software in Unreal Engine. These videos contained simulated walking along linear trajectories with constant head rotations in two environments with starts at random positions, varying environmental conditions, lighting, starting head poses and walking speed (see (*26*) for further details). Mineault

and colleagues demonstrated that DorsalNet units' activations in response to different visual stimuli can explain visual responses in a database of neural recordings along the primate dorsal stream better than many other computational models of motion processing.

In our study, we used the pre-trained DorsalNet model checkpoint provided by (*26*) for our simulated experiments ("airsim_dorsalnet_batch2_model.ckpt-3174400-2021-02-12 02-03-29.666899.pt"). We fed to the network drifting gratings and plaids moving in 12 equispaced directions at a fixed spatial and temporal frequency (0.0625 pixel^{-1} and 0.0625 frames^{-1} respectively). This allowed building direction tuning curves for grating and plaid responses and classify the units of the network as pattern or component, using the same criteria applied to neuronal recordings. We also fed spatiotemporally correlated noise movie, similar to those used in our neurophysiology experiments (again, spatial and temporal correlation was achieved by filtering withe noise frames with Gaussian filters and an exponential kernel). The spatial correlation scale (i.e., sigma of the Gaussian kernel) was chosen to have a full width at half maximum (FWHM) corresponding to half of the above-mentioned gratings period. This was done to ensure a good match between the spatial scale of gratings and plaids and the one of the filters inferred via STA. The time constant of the temporal kernel was chosen to have a FWHM of 1.5 frames, so as to boost the effectiveness of the noise movies in activating the units, without inducing strong temporal correlations in the inferred STA filters. Activations were sampled from all units at the center of the convolutional maps of all feature maps at the output layer of each block of the network (named layer # 1,2,3 … etc. in the main text), including the non-ResNet blocks (see Fig. 7C). This was done using the functions provided by (*26*) along with the pre-trained model.

Modeling the tuning of rat visual cortical neurons using the activations of DorsalNet units

The activations of DorsalNet units were also used to build models of the tuning of rat visual cortical neurons for the direction of the plaid and grating stimuli. We started from the representation matrices visualized as gray scale maps, in Fig. 8A and D. The same approach was applied in Fig. 9E for the pools of quasi-component (left) and quasi-pattern (right) unclassified cells (see main text). These N x M representation matrices were obtained by stacking pre-processed grating and plaid tuning curves as separate rows for each responsive neuron of a particular class (N=number of neurons, M=number of stimulus conditions). We adapted a standard methodology (*37–39*) to predict the responses of each neuron (i.e., each row) in these "target" matrices as a linear combination of the rows of similar "regressor" matrices that were built using the tuning curves of DorsalNet units (component, pattern or unclassified, pooling all the units belonging to the same class from every layer of the network, as in Fig 7H). The linear weights were obtained by minimizing the RMSE between measured and predicted tuning curves, using a leave-one-out cross-validation procedure over the 24 stimuli (i.e., columns of the matrices). This resulted in 24 train-test splits where the linear fit was performed on all stimuli but one (the "test" one), whose value was predicted using the trained model. By stacking all these cross-validated predictions in a matrix of the same size as the target one, we built the predicted representation matrices shown in Fig.8B-C and E-F. We used all 3 populations of DorsalNet units (i.e., component, pattern or unclassified) to predict all 3 populations of rat neurons. The average, cross-validated RMSE across neurons obtained for each set of DorsalNet regressors (as well as for the STA-based LN model) is reported in Fig.8G e Fig.9A. The fitting of the linear regression weights was performed with custom code using the Matlab function "fminunc" and an L2 regularization (with regularization parameter lambda=0.005).

DorsalNet-based reclassification of unclassified units

In an attempt to explore in depth the nature of unclassified cells, we reclassified rat neurons depending on which one of the three different models mentioned in the previous section (i.e. the ones using DorsalNet component, pattern or unclassified units as regressors) could best capture their tuning. The outcome of this re-classification is displayed in Fig. 9B and Fig. 9C, where the color inside the dots representing the neurons indicates which set of DorsalNet predictors achieved the lowest, cross-validated prediction error (i.e., RMSE). To better understand the extent to which the best-performing ("winner") set of regressors (i.e., those with the lowest RMSE) was really superior, as a predictor of the observed responses, as compared to the other two, we defined a "confidence score" of the reclassification, by computing its relative RMSE advantage with respect to the other models:

$$confidence\ score = \frac{|\ RMSE_{winner} - RMSE_{others}|}{RMSE_{others}}$$

$$RMSE_{winner} = min(\boldsymbol{RMSEs})$$

$$RMSE_{others} = \frac{1}{2}\ \left((\sum \boldsymbol{RMSEs}) - \ RMSE_{winner}\right)$$

Here $\boldsymbol{RMSEs}$ represents the vector storing the RMSEs obtained with the 3 models for a given neuron.

Estimation of the spatial frequency of the receptive fields yielded by the STA images

To obtain a quantitative estimate of the SF of a RF reconstructed via STA we followed the following procedure. For each neuron, we considered the "best" STA frame (i.e., the one with the highest contrast index CI). Only responsive neurons with "good" STA (i.e., highest contrast index ≥ 7.5) were included in this analysis. First, we isolated the strongest lobe of the RF, by identifying the sign of the most extremal z-score value that was present in the STA, then we set to zero all STA values with opposite polarity and we took the absolute value of the resulting STA image. Next, we fitted this pre-processed STA image with a 2D Gaussian model (using the "fminunc" function). Finally, we extracted the FWHM of the fitted Gaussian (by multiplying by 2.355 the average of σ_x and σ_y resulting from the Gaussian fit) and took it as the half period of the RF, from which we calculated the RF spatial frequency as SF = 1/period, after conversion from pixel to visual degrees. The resulting RF SFs are shown in fig. S1A.

Estimation of the RF boundaries

To measure the fraction of neurons with RFs that partially fell outside the stimulus display, thus intersecting its edges (despite our efforts to minimize such events during data collection; see Materials and Methods), we applied the following procedure. Given a unit, we fitted the absolute values of the "best" STA frame (i.e., the one highest contrast index) with a 2D Gaussian model (using the "fminunc" function). Again, only responsive neurons with "good" STA (i.e., highest

contrast index ≥ 7.5) were included in this analysis. Then, we took the FWHMs (derived from σ_x and σ_y) of the fitted 2D Gaussian to draw an ellipse that provided an estimate of the RF boundary. These are the RF boundaries that are shown in fig. S1C (dashed, if intersecting the edges of the stimulus display). Finally, we computed the fraction of ellipses intersecting the edges of the stimulus display for each sub-population (per area and cell type) as shown in fig. S1D.

Fig. S1

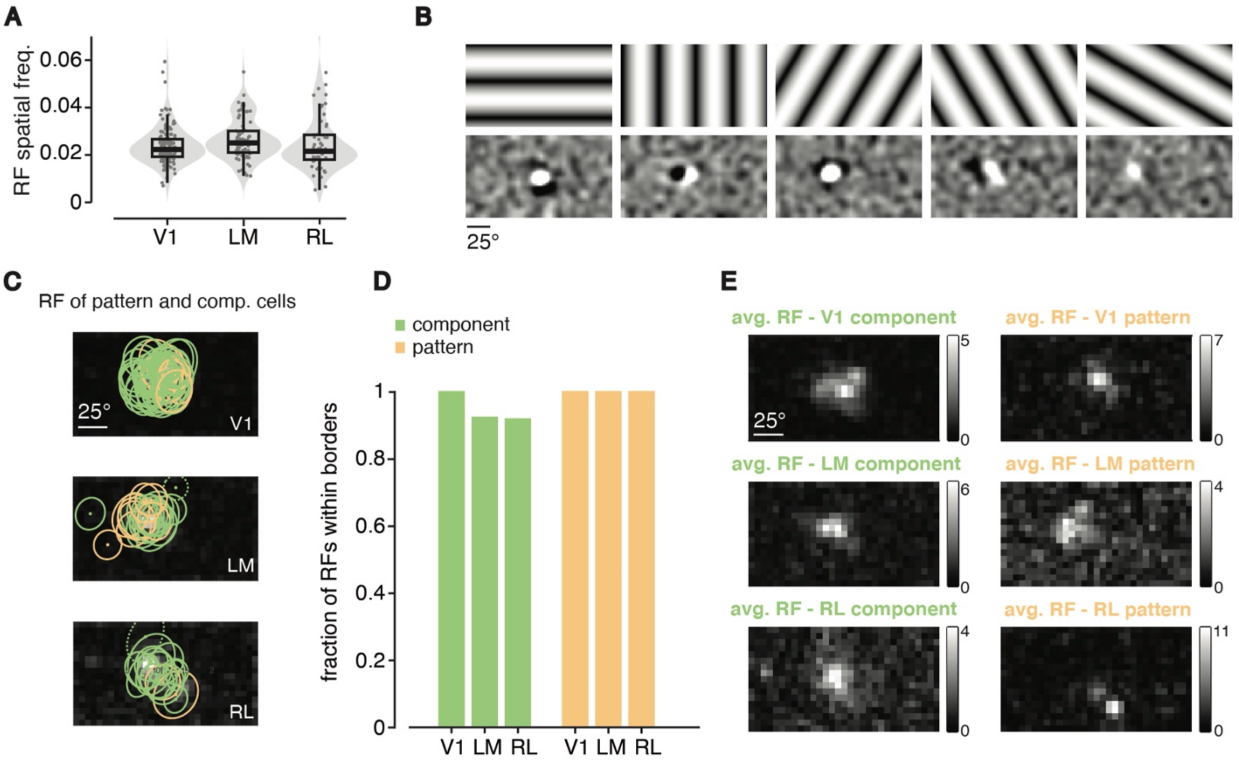

Fig. S1. **Spatial frequencies, positions and boundaries of the neuronal receptive fields recorded in visual areas V1, LM and RL.** (**A**) Spatial frequency distributions of the neuronal RFs, as computed from the STA images with the highest contrast obtained for the cells recorded in each visual area (see supplementary text). (**B**) The highest-contrast STA images obtained for some example cells are depicted along with the most effective 0.02 cpd grating for the unit. It can be noticed the good match between the shape of the RFs and the orientation/SF of the gratings. (**C**) RF profiles of the recorded units in each visual area (component and pattern cells are indicated, respectively, in green and orange). Each RF was estimated as the FWHM of the 2D Gaussian that best fitted the absolute values of the STA image (see supplementary text). The resulting ellipses were taken as the boundaries of the RFs. Those that intersected the edges of the stimulus display are plotted as dashed lines. (**D**) Bar plot depicting the fraction of component and pattern cells that were fully contained within the stimulus display (i.e., with RFs that did not intersect the edges of the display). (**D**) Population averages of the highest-contrast STA images obtained for pattern and component cells in the three visual areas (the gray scales report z-scored values).

Fig. S2.

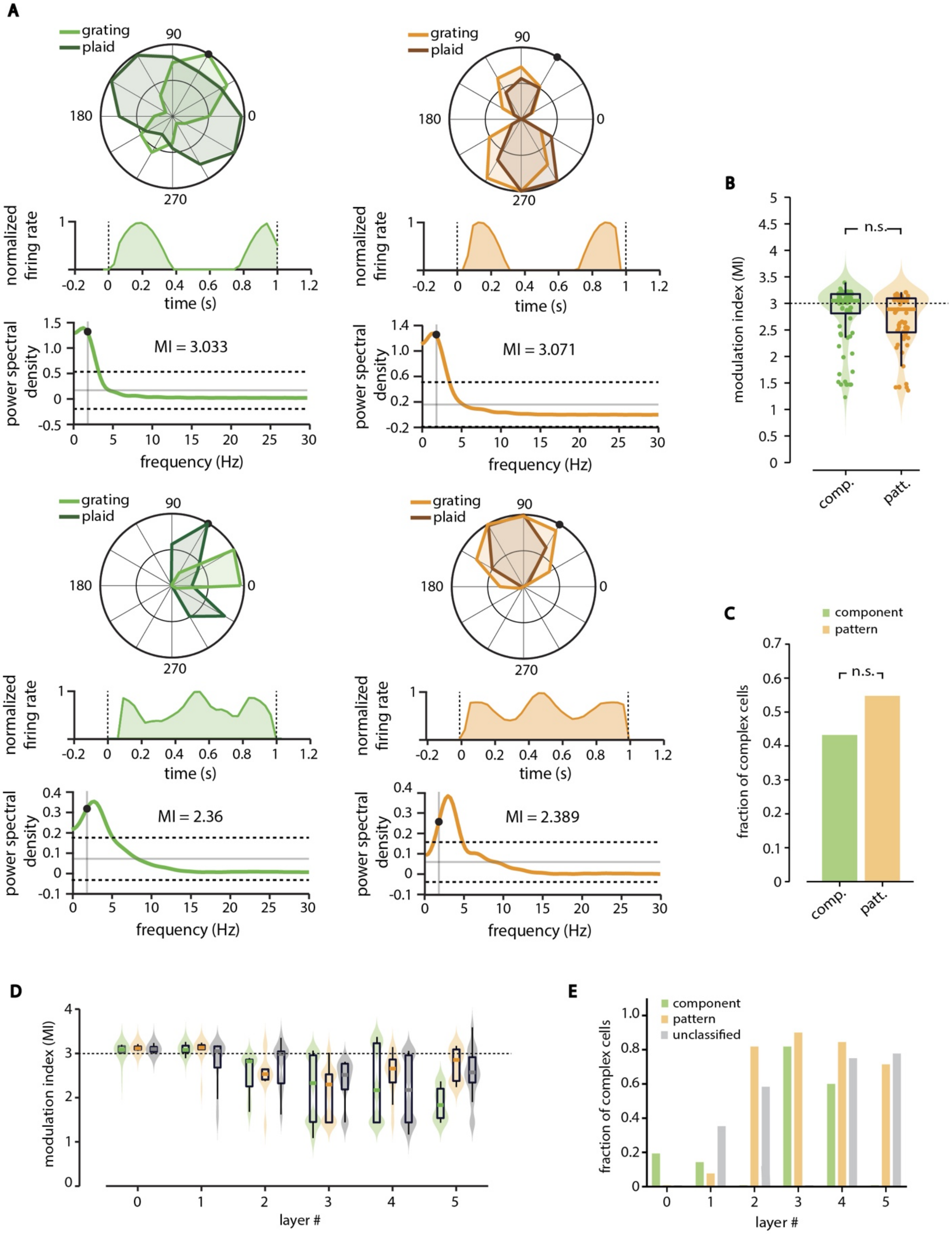
A
grating
plaid
90
180
0
270
normalized firing rate
time (s)
power spectral density
frequency (Hz)
MI = 3.033
MI = 3.071
MI = 2.36
MI = 2.389
B
modulation index (MI)
n.s.
comp.
patt.
C
component
pattern
fraction of complex cells
n.s.
comp.
patt.
D
modulation index (MI)
layer #
E
component
pattern
unclassified
fraction of complex cells
layer #

Fig. S2. **Phase invariance of component and pattern units in DorsalNet.** (**A**) Response dynamics of two example DorsalNet component units (left, green) and two example DorsalNet pattern units (right, orange), following presentation of the most effective grating. Each panel depicts: 1) the normalized tuning curves for gratings (light color) and plaids (dark color); 2) the activation profile of the unit during stimulus presentation; and 3) the power spectrum of the activation profile, with its mean (horizontal gray line), its mean ± SD (dashed lines), and the temporal frequency of the grating (vertical gray line; the black dot marks the value of the power spectrum at the stimulus frequency). The value of the MI (defined in the Materials and Methods) is also reported for each example unit. The two example units on the top display the highly-modulated response profile that is typical of "simple cells" (hence the high MI values), whereas the two example units on the bottom display a way more stable response profile, as typical of "complex cells" (hence the lower MI values). (**B**) Distributions of MI values for the populations of component (in green) and pattern (in orange) units, as computed from the response to the most effective grating for each unit. The dashed line indicates the conventional threshold to distinguish simple (MI > 3) from complex cells (MI < 3). Differently from what observed for rat visual neurons (see Fig. 6B), the median MI was not significantly higher for component than for pattern units ($p > 0.05$; Wilcoxon test). (**C**) Bar plot reporting the fraction of units in each population being classified as complex (i.e., having MI < 3). As for the recorded cortical cells (see Fig. 6C), this fraction was larger for pattern than for component units, but the difference did not reach statistical significance ($p > 0.05$; χ^2 test). (**D**) Distributions of MI values for the populations of component (in green), pattern (in orange) and unclassified (in gray) units across the layers of DorsalNet. (**E**) Bar plot reporting the fractions of component (in green), pattern (in orange) and unclassified (in gray) units being classified as "complex cells" (i.e., MI < 3) across the layers of the network.

Movie S1.

Movie S1. The aperture problem. Animated cartoon illustrating the “aperture problem”. The animation highlights the importance of component motion integration to enable unbiased perception of the global motion of extended objects, when observing the world through a small aperture (like the RFs of visual cortical neurons).
